# Diversification of *CLE* expression patterns and nonmeristematic roles for CLAVATA receptor‐like kinases in a moss

**DOI:** 10.1111/nph.70170

**Published:** 2025-05-06

**Authors:** Zoe Nemec‐Venza, George R. L. Greiff, C. Jill Harrison

**Affiliations:** ^1^ School of Biological Sciences University of Bristol Bristol BS8 1TQ UK; ^2^ Laboratoire Reproduction et Développement des Plantes, École Normale Supérieure de Lyon Lyon 69342 France

**Keywords:** CLAVATA, CLE, egg development, life cycle, *Physcomitrium patens*, sperm development, sporophyte

## Abstract

- The CLAVATA pathway controls meristematic cell proliferation and multiple nonmeristematic processes in Arabidopsis development. While CLAVATA ancestrally regulates meristematic proliferation in nonseed plant gametophytes, ancestral sporophytic and nonmeristematic functions in land plants are unknown.
- Here, we analysed the promoter activities of all peptide (*PpCLE*) and receptor‐encoding (*PpCLV1a*, *PpCLV1b* and *PpRPK2*) genes throughout the moss (*Physcomitrium patens*) life cycle and validated our expression analyses using mutant phenotype data.
- In gametophore apices, *PpCLE3* expression marked apical cells, and *PpCLV1b* and *PpRPK2* overlapped. In nonmeristematic tissues, gametophytes showed highly focal *PpCLE* but broader receptor‐encoding gene expression, and many genes were co‐expressed. Mutant phenotype analysis revealed roles for *PpCLV1a*, *PpCLV1b* and *PpRPK2* in fertility and male and female reproductive development. In sporophytes, no *PpCLE* expression specifically marked the apical cells, and *PpCLV1b* and *PpRPK2* expression initially marked distinct apical and basal domains, but later overlapped at the intercalary meristem. Overall, fewer genes were co‐expressed in sporophytes than in gametophytes, but all genes were co‐expressed in guard cells.
- Our data indicate that nonmeristematic CLAVATA functions in gametangium development and stomatal development may be ancestral within land plants. Peptide encoding (*CLE*) gene copy numbers amplified in mosses, and promoter evolution was a likely driver of cell type diversification during moss evolution.

## Introduction

Originally identified with roles in the Arabidopsis fruit and shoot apical meristem (Leyser & Furner, 1992; Clark *et al*., 1993), the CLAVATA pathway is a key signalling mechanism in plants (Fletcher, 2018). CLAVATA pathway genes encode CLE peptides that can diffuse between cells (Fletcher *et al*., 1999; Cock & McCormick, 2001; Rojo *et al*., 2002) and receptor‐like kinases such as CLAVATA1 and RECEPTOR‐LIKE PROTEIN KINASE2 (RPK2), which perceive the peptides and transduce a signal to downstream components (Clark *et al*., 1997; Trotochaud *et al*., 2000; Kinoshita *et al*., 2010). In the multicellular shoot apex of Arabidopsis, a CLAVATA signalling module comprising the CLAVATA3 (CLV3) peptide, the CLAVATA1 (CLV1) receptor‐like kinase and the downstream transcription factor, WUSCHEL, operate in a feedback loop to maintain the size and integrity of the meristem as the shoot grows (Mayer *et al*., 1998; Fletcher *et al*., 1999; Brand *et al*., 2000; Schoof *et al*., 2000). Modules with a similar logic but involving different CLEs, receptor‐like kinases and *WUSCHEL*‐like *WOX* genes are involved in cell type differentiation and proliferation in roots, the vascular cambium, developing stomata and the expanding leaf margin of Arabidopsis (Fletcher, 2020; Willoughby & Nimchuk, 2021; Narasimhan & Simon, 2022). Each gene class comprises many members (Goad *et al*., 2017; Whitewoods *et al*., 2018; Wu *et al*., 2019), which are expressed in diverse cell types and tissues (Jun *et al*., 2010; Fletcher, 2020; Tvorogova *et al*., 2021), and receptors can dimerise, adding complexity (Demesa‐Arevalo *et al*., 2024). Thus, Arabidopsis CLAVATA pathway components have meristematic and nonmeristematic functions.

*CLAVATA* genes originated in land plants, and previous studies comparing CLAVATA function between Arabidopsis and moss identified ancestral roles for CLAVATA in stem cell regulation (Whitewoods *et al*., 2018; Cammarata *et al*., 2022). Whereas CLAVATA signalling suppresses meristematic cell proliferation in moss (*Physcomitrium patens*) and Arabidopsis, it promotes such proliferation in fern (*Ceratopteris richardii*) and liverwort (*Marchantia polymorpha*) gametophytes (Whitewoods *et al*., 2018, 2020; Hirakawa *et al*., 2020; Cammarata *et al*., 2022; Renninger *et al*., 2024), and meristematic CLAVATA/WOX interactions are considered conserved to the level of euphyllophytes (Renninger *et al*., 2024) but not further (Sakakibara *et al*., 2014; Hirakawa *et al*., 2020). Nonmeristematic roles for CLAVATA were recently identified in *C. richardii,* in which *CrCLV3* expression is upregulated in developing antheridia, and *Crclv3kd* plants produce fewer antheridia than wild‐type (WT) plants and show a delay in archegonium maturation (Renninger *et al*., 2024). However, nonmeristematic functions have not been identified in other nonseed plants. Thus, the pattern of diversification of meristematic and nonmeristematic CLAVATA functions in land plants is currently unclear, with knowledge gaps in each major group.

Bryophytes have undergone significant gene loss during their divergence from the last common ancestor of land plants (Clark, 2023). However, the moss CLAVATA pathway comprises many more components than liverwort or hornwort pathways, suggesting potential for lineage‐specific functional diversification (Whitewoods *et al*., 2018, 2020). While moss tissues typically have fewer cell layers than flowering plant tissues, mosses have diverse cell types in both life cycle stages (Parihar, 1959; Kofuji & Hasebe, 2014). Single‐celled haploid spores germinate to establish apical cells that cleave to develop filamentous protonemal tissues comprising photosynthetic chloronemata and foraging caulonemata (Parihar, 1959). Both filament types initiate side branches, and in caulonemata, *c*. 5% of these attain bud identity, initiating shoot‐like gametophores with leaf‐like phyllids (Aoyama *et al*., 2012). Male and female sex organs (antheridia and archegonia) differentiate at the tip of gametophores, respectively, producing sperm and eggs (Parihar, 1959). Fertilization results in diploid zygote formation, embryogenesis and sporophyte development, and sporophyte development terminates in sporangium formation and spore production, completing the life cycle (Parihar, 1959).

*Physcomitrium patens* has nine *CLE*s (*PpCLE1‐9*), which together encode four CLV3‐like peptides, and four receptor‐like kinases (PpCLV1a, PpCLV1b, PpRPK2 and PpCR4) have been implicated in peptide signalling (Whitewoods *et al*., 2018, 2020; Cammarata *et al*., 2022; Shumbusho *et al*., 2024). Previous work identified roles for CLAVATA in protonemal development and gametophore development (Whitewoods *et al*., 2018, 2020; Cammarata *et al*., 2022; Nemec‐Venza *et al*., 2022; Shumbusho *et al*., 2024). During protonemal development, most *CLAVATA* promoters are active in caulonemal tip cells, and pharmacological experiments and expression analyses in *Ppclv1a1b* and *Pprpk2* mutant backgrounds suggest that *PpCLV1a/PpCLV1b* and *PpRPK2* suppress caulonemal identity by promoting polar auxin transport out of filament tip cells by PIN‐FORMED1 (PIN) proteins (Nemec‐Venza *et al*., 2022). Later in the life cycle, mutants show defects in gametophore initiation, having supernumerary but frequently abortive gametophores (Whitewoods *et al*., 2018, 2020). In *Ppclv1b, Ppclv1a1b* and *Pprpk2* mutants, differentiation at the gametophore base is perturbed, and stem cells are overproduced, but peptide application can suppress cell proliferation in phyllids (Whitewoods *et al*., 2018, 2020; Cammarata *et al*., 2022). Modelling and combinatorial mutant phenotype analysis showed that *PpCLV1a*/*PpCLV1b* and *PpRPK2* act independently via unknown downstream components to suppress gametophore stem cell identity and that *PpCLV1a*/*PpCLV1b* repress cytokinin‐mediated stem cell initiation (Cammarata *et al*., 2022).

To explore the evolution of meristematic and nonmeristematic CLAVATA functions in land plants, we analysed the expression of peptide and receptor‐encoding genes throughout the *P. patens* life cycle and examined reproductive phenotypes in receptor mutants. We found that all the tissues described previously exhibited promoter activity of at least one *PpCLE* and receptor‐like kinase gene, with receptor‐encoding genes typically having broader domains of promoter activity than *PpCLE*s. While all *PpCLE* promoters were active during caulonema and phyllid development, promoter activity was spatiotemporally restricted at other stages of gametophyte development, frequently marking a single cell type within a given tissue. Several peptide and receptor‐encoding genes were expressed in developing gametangia, and receptor mutants had defects in fertility and antheridium, archegonium, sperm and egg development. In sporophytes, *PpCLV1b* and *PpRPK2* expression was in mutually exclusive domains during early embryogenesis but later overlapped in intercalary meristems, and all *PpCLE* and receptor promoters were active in stomata. Overall, our data pinpoint multiple foci of CLAVATA signalling at different sites throughout the *P. patens* life cycle and identify potential conservation in CLAVATA function between bryophytes and vascular plants in gametangium formation and stomatal development.

## Materials and Methods

### Plant material and growth conditions

The Gransden strain of *P. patens (Hedw.) Mitt*. was used as the WT strain for all experiments, and *Ppclv1a*, *Ppclv1b*, *Ppclv1a1b*, *Pprpk2*, *Ppclv1a1brpk2*, *PpCLE1::NGG*, *PpCLE2::NGG*, *PpCLE3::NGG, PpCLE4::NGG, PpCLE5::NGG, PpCLE6::NGG, PpCLE7::NGG*, *PpCLE8::NGG, PpCLE9::NGG, PpCLV1a::NGG*, *PpCLV1b::NGG* and *PpRPK2::NGG* and mutant line generation strategies were previously described (Whitewoods *et al*., 2018, 2020; Cammarata *et al*., 2022; Nemec‐Venza *et al*., 2022). With the exception of *PpCLV1a::NGG*, in which expression was analysed in three lines due to sporophyte development defects, a single reporter line was used for each gene.

For vegetative expression analyses, plants were spot propagated on BCDAT media containing 0.5% agar, as in a previous study (Nemec‐Venza *et al*., 2022) and grown at 23°C in continuous light or at 22°C in long‐day conditions (16 h : 8 h, light : dark). To induce sexual reproduction, protonemal homogenates were propagated in Magenta vessels containing hydrated peat plugs with 30 ml of half‐strength BCD media and grown at 23°C in continuous light for 8–10 wk before transfer to 16°C short‐day conditions (16 h : 8 h, dark : light). To stain gametangia and phenotype plants, tissue was harvested 2–3 wk after induction. At this point, sterile water was repeatedly poured on the tissue until it was wet enough to allow fertilization. To stain young and mature sporophytes from *promoter::NGG* lines, tissue was respectively collected 12 d and 24 d after fertilization. For the receptor mutant lines, cultures were maintained for 2 months following fertilization to determine sporophyte production rates.

### Histochemical analysis and imaging

Beta‐glucoronidase (GUS) activity was assayed at 37°C with a solution containing 100 mM phosphate buffer (pH 7.0), 10 mM Tris–HCl (pH 8.0), 1 mM ethylenediaminetetraacetic acid, 0.05% Triton X‐100, 2 mM potassium ferricyanide, 2 mM potassium ferrocyanide and 1 mg ml^−1^ X‐Gluc (5‐bromo‐4‐chloro‐3‐indolyl‐β‐d‐glucuronic acid) dissolved in 10% (v/v) dimethylsulfoxide. Staining times for buds and leaves were as follows: 7.5 h for *PpCLE3::NGG*, *PpCLE4::NGG*, *PpCLE5::NGG*, *PpCLE6::NGG*, *PpCLV1b::NGG* and *PpRPK2::NGG*; 15 h for *PpCLE2::NGG*, *PpCLE8::NGG*, *PpCLE9::NGG* and *PpCLV1a::NGG*; and 21 h for *PpCLE1::NGG* and *PpCLE7::NGG*. Antheridia, archegonia and sporophyte staining times are indicated in figures or figure legends. After staining, samples were cleared in 70% ethanol for 24 h. Imaging was performed using a Keyence VHX‐1000 digital microscope (whole gametophores and leaves), a Leica DM2000 microscope (buds) or a Zeiss AxioImager.M2 microscope (antheridia, archegonia and sporophytes). For green fluorescent protein (GFP) imaging, confocal images were acquired with a Zeiss LSM 980 microscope using either a 4× or a 20× objective. Automatic excitation and emission settings were used to visualize GFP and propidium iodide staining in two separate channels.

### Mutant phenotyping

Wild‐type, *Ppclv1a*, *Ppclv1b*, *Pprpk2*, *Ppclv1a1b* and *Ppclv1abrpk2* mutants were grown in reproduction‐inducing conditions. Gametangia (antheridia and archegonia) were dissected from the fresh apices, and quantification of gametangium morphology was performed using at least two experimental replicates. To assess fertility, sporophytes from three experimental replicates were counted 2 months after fertilization. A GXML 1500 compound microscope was used to image tissues, and the data were subsequently analysed in imagej. Statistical analysis was performed using the R Statistical software (v.4.1.2) (R Core Team, 2021). To analyse phyllid phenotypes, dissected gametophores were cleared with Hoyer's medium overnight, washed in water and soaked in 2 M NaOH for 2 h (Dennis *et al*., 2019). After another washing step, samples were stained in 0.05% toluidine blue for 90–120 s and then washed again. Phyllids were then dissected and mounted for imaging. Mutant phenotype comparisons used phyllid 9 in the heteroblastic series from *Ppclv1a* and *Ppclv1b* mutants and WT plants (Barker & Ashton, 2013). Phyllid half area and cell number were calculated as described previously (Dennis *et al*., 2019).

### Gamete imaging

To visualize gametes, whole gametophores were cleared and mounted in Hoyer's solution (50 ml of distilled water, 30 g gum arabic, 200 g chloral hydrate and 20 ml of glycerin) and rinsed in water. To visualize sperm cells, antheridia were then dissected and stained with 1 mg l^−1^ 4', 6‐diamidino‐2‐phenylindole (DAPI) as detailed in Horst & Reski (2017). To visualize egg cells, differential interference contrast imaging was used. All imaging was performed with a Zeiss AxioImager.M2 epifluorescence microscope.

### Correlation analysis

Dendrograms were based on Pearson's correlation coefficient as calculated by cor() and generated based on Euclidean distance with the hclust() function using the ‘average’ method with the R Statistical software (v.4.1.2) (R Core Team, 2021). The corrplot() function was used to generate correlation plots.

## Results

### *PpCLE3* , *PpCLV1b* and *PpRPK2* promoters were active in gametophore buds

**Fig. 1:**
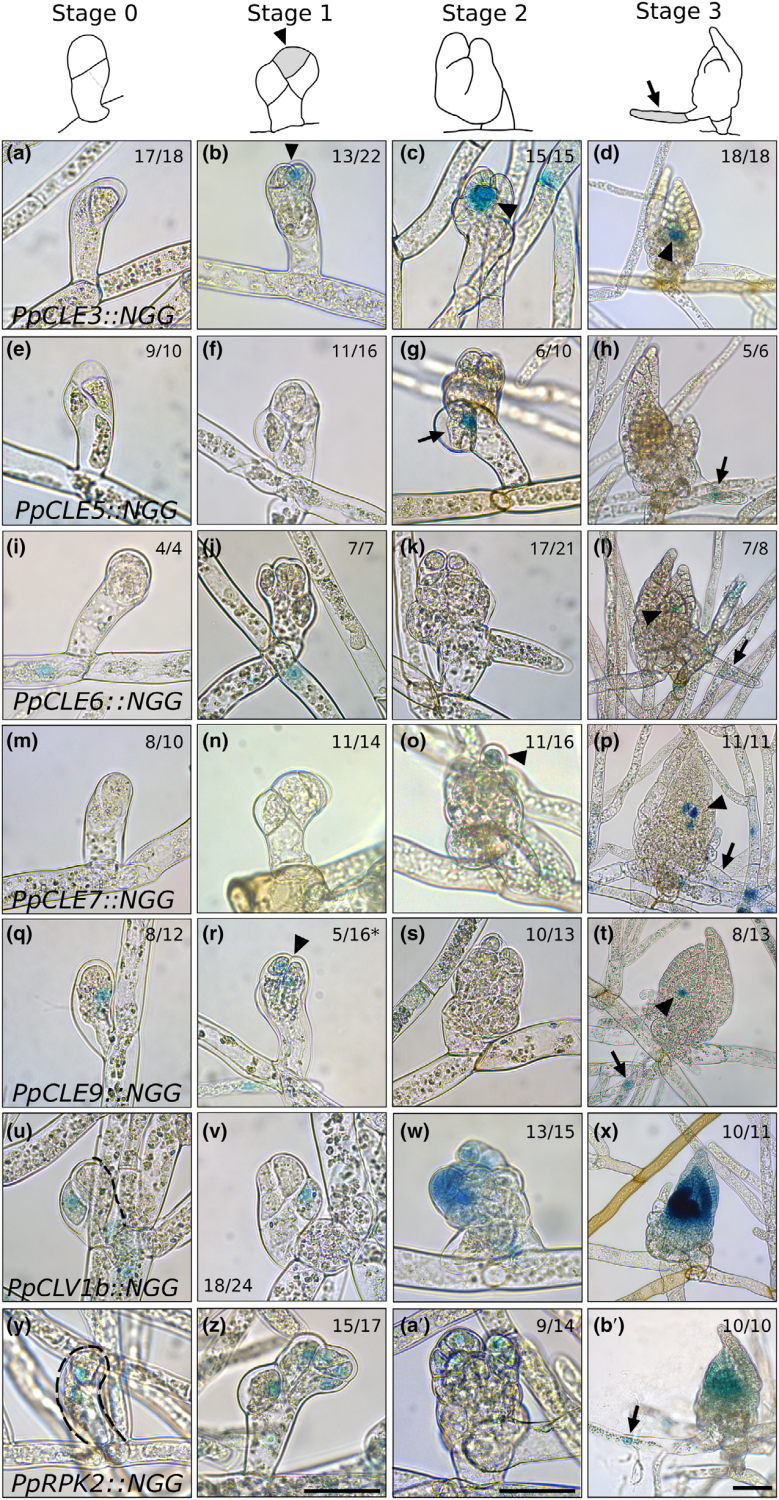
*CLAVATA* promoters were active during gametophore initiation in *Physcomitrium patens*. (a–d) *PpCLE3::NGG* activity at (a) Stage 0, (b) Stage 1, (c) Stage 2 and (d) Stage 3 of gametophore development. Activity was undetectable at Stage 0 but marked apical cells from Stage 1 and throughout subsequent development. (e–h) *PpCLE5::NGG* activity at (e) Stage 0, (f) Stage 1, (g) Stage 2 and (h) Stage 3 of gametophore development. Activity marked rhizoid initiation and subsequent development. (i–l) *PpCLE6::NGG* activity at (i) Stage 0, (j) Stage 1, (k) Stage 2 and (l) Stage 3 of gametophore development. Activity was detected in cells underlying initiating buds and at the apex and rhizoid tips at Stage 3. (m–p) *PpCLE7::NGG* activity at (m) Stage 0, (n) Stage 1, (o) Stage 2 and (p) Stage 3 of gametophore development. *PpCLE7::NGG* expression was detected in apical hairs and rhizoids. (q–t) *PpCLE9::NGG* activity at (q) Stage 0, (r) Stage 1, (s) Stage 2 and (t) Stage 3 of gametophore development. Activity was detected in two‐celled buds, the apical portion of a minority of Stage 1 buds and rhizoids and phyllid initials in Stage 3 buds. (u–x) *PpCLV1b::NGG* activity at (u) Stage 0, (v) Stage 1, (w) Stage 2 and (x) Stage 3 of gametophore development. Activity was detected at the base of two‐ or four‐celled buds but was stronger in phyllid primordia and the apex from Stage 2. (y–b′) *PpRPK2::NGG* activity at (y) Stage 0, (z) Stage 1, (a') Stage 2 and (b') Stage 3 of gametophore development. *PpRPK2* promoter activity was detected throughout buds and was later detected in apices, phyllid primordia and rhizoids. Arrowheads indicate expression in the apical region; arrows indicate rhizoid expression. Numbers indicate the proportion of buds with a similar expression pattern. Asterisks indicate a minority of buds were stained. Bars: (Stages 0–2) 50 μm; (Stages 3–4) 100 μm.

To resolve the location of *CLAVATA* activity and likely peptide/receptor‐like kinase signalling at different lifecycle stages, we first used *promoter::nuclearGFP‐GUS* (*pro::NGG*) fusions to characterize the patterns of promoter activity during gametophore bud development (Fig. 1). We defined morphological markers for each stage of bud development: Stage 0 buds had two or three cells; Stage 1 buds had 4–8 cells and a wedge‐shaped apical cell; Stage 2 buds had tetrahedral apical cells, visible phyllid primordia and initiating rhizoids; Stage 3 buds had a pointed shape with two or three phyllid primordia covering the apex (Fig. 1); and Stage 4 buds had two to three fully expanded leaves (Supporting Information Figs S1, S2). All *promoter::NGG* lines except for *PpCLE1::NGG*, *PpCLE8::NGG* and *PpCLV1a::NGG* lines accumulated signal in a range of tissues in and/or around initiating buds (Figs 1, S1, S2). At Stage 0, staining was most conspicuous in *PpCLV1b::NGG* and *PpRPK2::NGG* lines and detected in buds (Fig. 1u,y). *PpCLE6::NGG* activity was observed in bud mother cells (Fig. 1i), and *PpCLE9::NGG* activity was observed in Stage 0 buds (Fig. 1q). No other lines showed conspicuous staining (Figs 1a,e,m, S1). At Stage 1, apical staining was observed in *PpCLE3::NGG* (Fig. 1b), *PpRPK2::NGG* (Fig. 1z) and some *PpCLE9::NGG* buds (Fig. 1r), while lateral staining was observed in *PpCLV1b::NGG* buds (Fig. 1v). Notably, *PpCLE3::NGG* activity marked the apical cell or the apical cell and close daughters throughout subsequent stages of bud development (Figs 1b–d, S1, S2). Staining was usually absent in *PpCLE5::NGG* and *PpCLE7::NGG* lines (Figs 1f,n, S1). From Stage 2, *PpCLE5::NGG* activity was strong and specific to rhizoid precursor cells and rhizoid tips (Figs 1g,h, S2), while *PpCLE2::NGG* (Fig. S1), *PpCLE6::NGG* (Fig. S1), *PpCLE7::NGG* (Fig. 1p), *PpCLE9::NGG* (Fig. 1t) and *PpRPK2::NGG* (Fig. 1b′) activities were observed later during rhizoid development. *PpCLE7::NGG* (Figs 1o,p, S2) activity was observed in the axillary hairs. From Stage 3, *PpCLE4::NGG* (Fig. S1), *PpCLE6::NGG* (Fig. 1l) and *PpCLE9::NGG* (Fig. 1t) promoter activities were detected in single cells close to the apex, and this pattern was maintained at Stage 4 (Fig. S1). *PpCLV1b::NGG* (Fig. 1w,x) and *PpRPK2::NGG* (Fig. 1a′,b′) activities marked phyllid primordia and the whole apical region from Stage 2 to Stage 4 (Fig. S1). Taken with mutant phenotype analyses, which showed that CLAVATA suppresses leafy shoot initiation and stem cell proliferation but is required for correct 3D growth determination (Whitewoods *et al*., 2018, 2020; Cammarata *et al*., 2022), these distinct patterns of peptide and receptor‐encoding gene promoter activity suggest a potential role for short‐range signalling in the establishment of the apical‐basal bud axis, the orientation of division planes in the bud and gametophore patterning. We postulate that *PpCLE6, PpCLE9*, *PpCLV1b* and *PpRPK2* are the most likely candidates to control Stage 0 bud cell division planes and that *PpCLE3* is likely to act with *PpCLV1b* and *PpRPK2* to regulate apical stem cell function and/or early phyllid development.

### All promoters were active in gametophores

**Fig. 2:**
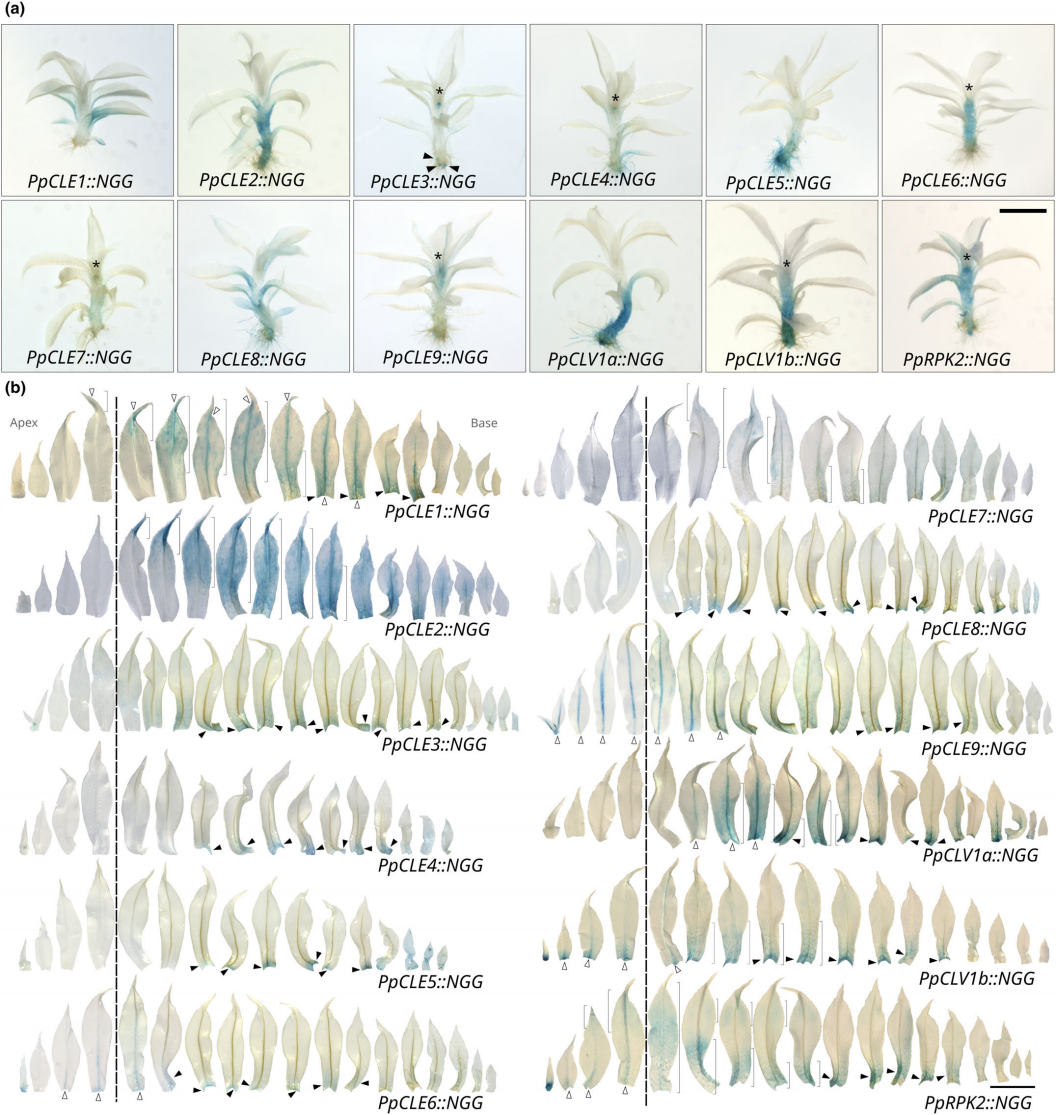
*CLAVATA* promoters were active during gametophore development. (a) In *Physcomitrium patens* gametophores, *PpCLE3::NGG*, *PpCLE4::NGG*, *PpCLE6::NGG*, *PpCLE7::NGG*, *PpCLE9::NGG, PpCLV1b::NGG* and *PpRPK2::NGG* lines showed activity around the apical region (asterisk), and signal in *PpCLE3::NGG* lines was also observed in the apical cells of branches initiating at the gametophore base (arrowheads). *PpCLE2::NGG*, *PpCLE6::NGG*, *PpCLV1a::NGG*, *PpCLV1b::NGG* and *PpRPK2::NGG* promoter activities were detected in the gametophore axis, and *PpCLE5::NGG* was active in rhizoids from early emergence. Phyllid staining was observed in all lines. (b) At early stages of phyllid development (P1–P4 to the left of the dashed lines), *PpCLE6::NGG* activity was detected in the basal portion of the midrib, and *PpCLE9::NGG* was active in the whole midrib. *PpCLV1b::NGG* and *PpRPK2::NGG* activities were detected in the basal portion of the lamina and midrib. In fully expanded phyllids (P5 or more to the right of the dashed lines), basipetal waves *of PpCLE1::NGG*, *PpCLE2::NGG*, *PpCLE7::NGG*, *PpCLV1a::NGG*, *PpCLV1b::NGG* and *PpRPK2::NGG* activities were detected. Black arrowheads indicate staining localized at the leaf base, white arrowheads indicate staining in the midvein, and brackets indicate basipetal waves of gene expression. At least three phyllid series were imaged from each line with replicates showing similar results. Bars, 1 mm.

To further investigate roles for *CLAVATA* in gametophore and phyllid development, we stained and imaged gametophores from the lines previously described and analysed phyllid phenotypes in receptor mutants (Figs 2, S2, S3). While *PpCLE3*, *PpCLE4*, *PpCLE6, PpCLE7* and *PpCLE9* promoters were persistently active in gametophore apices (Fig. 2a), *PpCLE3::NGG* activity marked apical cells and branch meristems initiating at the gametophore base, whereas apical *PpCLE9::NGG* activity was broader (Fig. 2a). *PpCLV1b::NGG* and *PpRPK2::NGG* also showed activity in the apical region (Fig. 2a). *PpCLE6::NGG*, *PpCLV1a::NGG*, *PpCLV1b::NGG* and *PpRPK2::NGG* showed activity in the gametophore axis (Fig. 2a), and *PpCLE5::NGG* activity was strongest in rhizoid development, as in buds (Fig. 2a). To some extent, all promoters were active during phyllid development with the stage varying by promoter, and most promoters showed activity at the phyllid base throughout development (black arrowheads in Fig. 2b). In expanding phyllids (P1–P4, to the left of the dashed line), *PpCLE6::NGG* activity was observed at the midrib base, *PpCLE9::NGG* activity marked the midrib, and *PpCLV1b::NGG* and *PpRPK2::NGG* activity marked the base in both the midrib and the lamina (white arrowheads in Figs 2b, S2). As or after phyllids attained their maximum length (P5 or older, to the right of the dashed line), a wave of promoter activity proceeding from phyllid tips to bases was observed in *PpCLE1::NGG*, *PpCLE2::NGG*, *PpCLE7::NGG* and *PpRPK2::NGG* lines (brackets in Fig. 2b). Similar waves of promoter activity started from the middle portion of the lamina in *PpCLV1a::NGG* and *PpCLV1b::NGG* lines and were also observed in the midrib of *PpCLE1::NGG* and *PpCLV1a::NGG* lines (brackets and white arrowheads in Fig. 2b). *PpCLV1a::NGG*, *PpCLV1b::NGG* and *PpRPK2::NGG* activities persisted at the base of fully developed phyllids (Fig. 2b). *PpCLE3::NGG*, *PpCLE4::NGG*, *PpCLE5::NGG* and *PpCLE8::NGG* activities were more pronounced in juvenile phyllids (far right of Fig. 2b). Analysis of receptor mutant phenotypes showed that *PpRPK2* has a role in phyllid shape determination as mutant phyllids have irregular cell division planes and laminar outgrowths in multiple orientations. While *Ppclv1a* mutants had no obvious abnormalities, *Ppclv1b* mutants had fewer cells than WT phyllids, and thus *PpCLV1b* promotes phyllid cell proliferation (Fig. S3). Thus, *CLAVATA* promoter activity was observed in a range of gametophore tissues, with varying degrees of cell type specificity, and CLAVATA can suppress (Whitewoods *et al*., 2018) or promote cell proliferation in phyllids depending on the level of peptide availability (Fig. S3). *PpCLE3* promoter activity, *PpCLE5* promoter activity and *PpCLE9* promoter activity were respectively strongly elevated in apical cells, rhizoids and the midrib of expanding phyllids relative to other cell and tissue types (Figs 1, 2, S2).

### *PpCLV1a* , *PpCLV1b* and *PpRPK2* regulate fertility

**Fig. 3:**
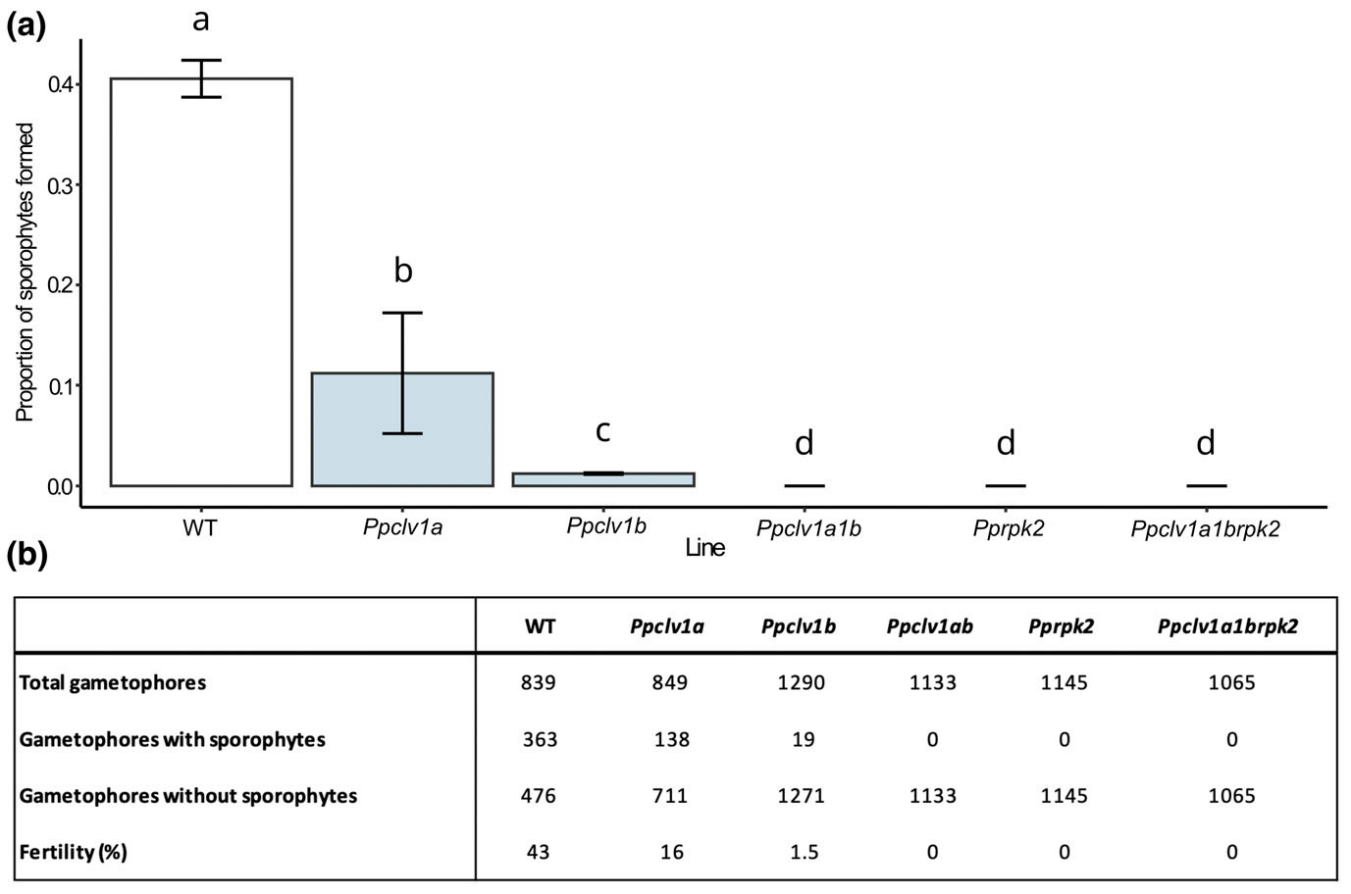
Fertility impairment in *clavata* mutants. (a) Bar chart comparing the proportion of gametophores with or without sporophytes in *Physcomitrium patens* wild‐type (WT) and mutant plants after growth in inductive conditions. Different letters indicate significant differences between groups identified by ANOVA and Tukey test. The error bars represent standard errors of the mean values. (b) Table showing frequency of sporophyte development in WT and mutant plants. Pooled data from at least two experimental replicates were used. *P* < 0.05.

Patterns of promoter activity shown in Figs 1 and 2 help to account for defective bud and gametophore development mutant phenotypes previously reported (Whitewoods *et al*., 2018, 2020; Cammarata *et al*., 2022). However, previous work did not investigate roles for the *P. patens* CLAVATA pathway in reproductive development. During the reproductive phase transition, archegonia (egg‐producing female gametangia) and antheridia (sperm‐producing male gametangia) are produced at the shoot apices. Moisture triggers the rupture of sac‐like antheridia and sperm cell release, and sperm are attracted to mature archegonia. The basal cavity of each archegonium contains an egg cell, and sperm cells from the same or nearby plants swim through the hollow archegonium neck to reach the egg. Fertilization results in diploid zygote formation, and this is followed by embryo and sporophyte development (Parihar, 1959). Our first investigation into potential roles for CLAVATA in reproductive development comprised a fertility analysis in WT vs receptor mutant plants through selfing (Fig. 3). This showed that *Ppclv1a1b*, *Pprpk2* and *Ppclv1a1brpk2* mutant plants were sterile (Fig. 3a). While 43% of WT gametophores produced sporophytes, only 16% of *Ppclv1a* and 1.5% of *Ppclv1b* gametophores produced sporophytes (Fig. 3b). Thus, *PpCLV1a and PpCLV1b* are jointly required for fertility, with *PpCLV1b* playing a greater role, and *PpRPK2* may act independently or together with *PpCLV1a/PpCLV1b*.

### *PpCLE6* , *PpCLE9* , *PpCLV1a* and *PpRPK2* promoters were active in antheridia

**Fig. 4:**
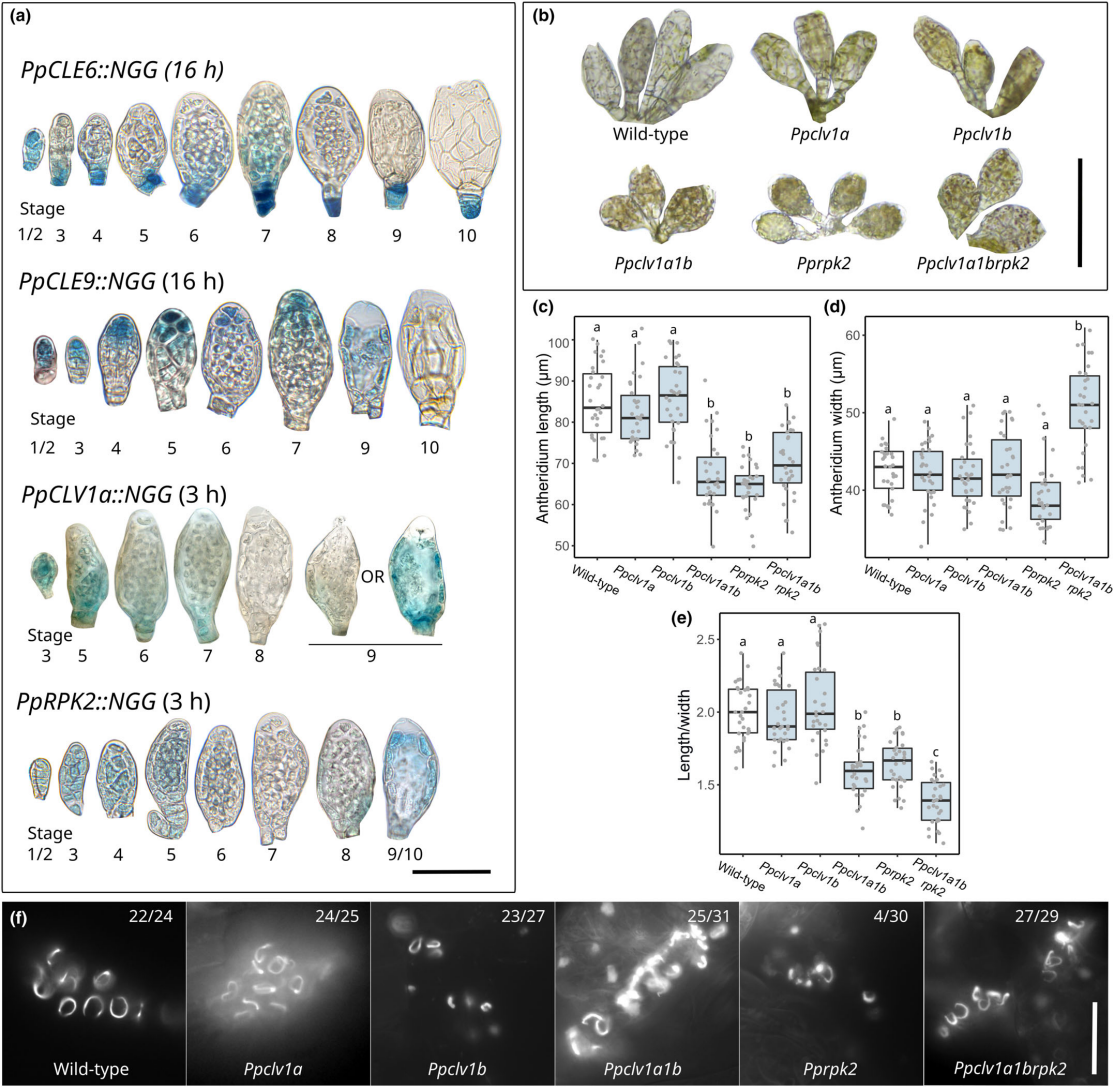
CLAVATA regulates male reproductive development. (a) Light micrographs of Beta‐glucoronidase (GUS)‐stained and dissected antheridia from *Physcomitrium patens* showing *PpCLE6::NGG*, *PpCLE9::NGG, PpCLV1a::NGG* and *PpRPK2::NGG* activities at different stages of development. While *PpCLE6::NGG* and *PpCLV1a::NGG* activities were mainly basal, *PpCLE9::NGG* activity was mainly apical, and *PpRPK2::NGG* activity extended throughout developing antheridia. Staining was less intense at Stage 8 and Stages 6 to 8 in *PpCLV1a::NGG* and *PpRPK2::NGG* lines. Only some *PpCLV1a::NGG* antheridia at Stage 9 showed expression. Staining times are displayed in brackets. Bar, 50 μm. (b) Light micrographs of antheridia dissected from gametophore apices, showing developmental defects in *Ppclv1a1b*, *Pprpk2* and *Ppclv1a1brpk2* mutants. Bar, 100 μm. (c) Boxplot showing that *Ppclv1a1b*, *Pprpk2* and *Ppclv1a1brpk2* mutants had shorter antheridia than wild‐type (WT) and *Ppclv1a* and *Ppclv1b* mutant plants. *n* > 30. (d) Boxplot showing that *Ppclv1a1brpk2* mutants had wider antheridia than WT plants. *n* > 30. (e) Boxplot showing that *Ppclv1a1b, Pprpk2* and *Ppclv1a1brpk2* mutants had more globose antheridia than WT plants *n* > 30. In all graphs, mid‐lines represent median values, boxes represent the 1^st^ to 3^rd^ quartile range, whiskers represent 95% confidence interval. Different letters indicate significant differences between groups identified by ANOVA and Tukey test. (f) Fluorescence micrographs of mature 4', 6‐diamidino‐2‐phenylindole (DAPI)‐stained sperm from WT and receptor mutant plants showing sperm with normal morphology. The numbers at the top right of each panel indicate the proportion of closed antheridia containing sperm cells. Bar, 20 μm. *P* < 0.05.

To identify the developmental basis of the fertility defects described previously, we next analysed patterns of peptide and receptor gene promoter activity and receptor mutant phenotypes in developing gametangia, starting with antheridia (Fig. 4). Antheridial development was previously staged by Landberg *et al*. (2013), who identified formative cell divisions during Stages 1–5 of development and sperm cell maturation from Stages 6 to 9 of development. Sperm cells are initially round (Stages 6 and 7) before elongating to become filiform (Stages 8 and 9), and sperm cells are released as antheridia open at Stage 9; an empty antheridial sac comprises Stage 10 (Landberg *et al*., 2013). While *PpCLE2::NGG*, *PpCLE7::NGG*, and *PpCLV1b::NGG* promoter activities were undetectable during antheridium development, *PpCLE1::NGG*, *PpCLE3::NGG, PpCLE4::NGG*, *PpCLE5::NGG* and *PpCLE8::NGG* lines showed weak staining in a minority of antheridia at Stages 6 and 7 (Fig. S4). The *PpCLE6* promoter was active at all developmental stages, showing strong staining in the antheridial stalks (Fig. 4a). Conversely, the *PpCLE9* promoter was active in the apical portion of antheridia, with more intense staining in the apical cell that later ruptures to allow antheridial opening. Basal *PpCLV1a::NGG* activity was detected up to Stage 7 of antheridium development, and activity was later observed throughout a minority of Stage 9 samples (Fig. 4a). *PpRPK2::NGG* activity was observed throughout the Stages 1–5 antheridium, with less intensity at Stages 6/7 and then more intensity at Stages 9/10 (Fig. 4a). Overall, these staining patterns suggest the presence of apical (*PpCLE9*) and basal (*PpCLE6*) sources of CLE peptides during antheridium development, with likely perception by PpCLV1a and/or PpRPK2 receptors.

### *PpCLV1a* , *PpCLV1b* and *PpRPK2* regulate male reproductive development

To investigate CLAVATA receptor functions in antheridium development and validate our expression data, we induced reproduction in *Ppclv1a*, *Ppclv1b*, *Ppclv1a1b*, *Pprpk2* and *Ppclv1a1brpk2* mutants and compared the morphology of fully developed WT and mutant antheridia (Fig. 4b). While gross antheridium structure appeared normal in mutants, *Ppclv1a1b, Pprpk2* and *Ppclv1a1brpk2* mutant antheridia were significantly shorter than WT antheridia, and *Ppclv1a1brpk2* antheridia were wider than WT antheridia (Fig. 4c,d). *Ppclv1a1b, Pprpk2* and *Ppclv1a1brpk2* mutant antheridia were smaller and more globose than WT antheridia (Fig. 4e). Although sperm cells with normal morphology were ultimately recovered from all mutant lines, the vast majority of *Pprpk2*, but not other mutant antheridia, were found empty (Fig. 4f). On one occasion, we were able to use an R2D2 reporter (Thelander *et al*., 2019) as a sperm donor to cross into *Ppclv1a1b* but not *Pprpk2* mutant plants (data not shown). Thus, antheridial defects were present in all sterile mutants; defects in male reproductive development contribute to fertility impairment in *Ppclv1a1b* and *Pprpk2* mutants (Figs 3, 4), and *PpCLV1a* and *PpCLV1b* are epistatic to *PpRPK2* with respect to sperm development (Fig. 4f).

### *PpCLE3* , *PpCLE4* , *PpCLE9* , *PpCLV1a* , *PpCLV1b* and *PpRPK2* promoters were active in archegonia

**Fig. 5:**
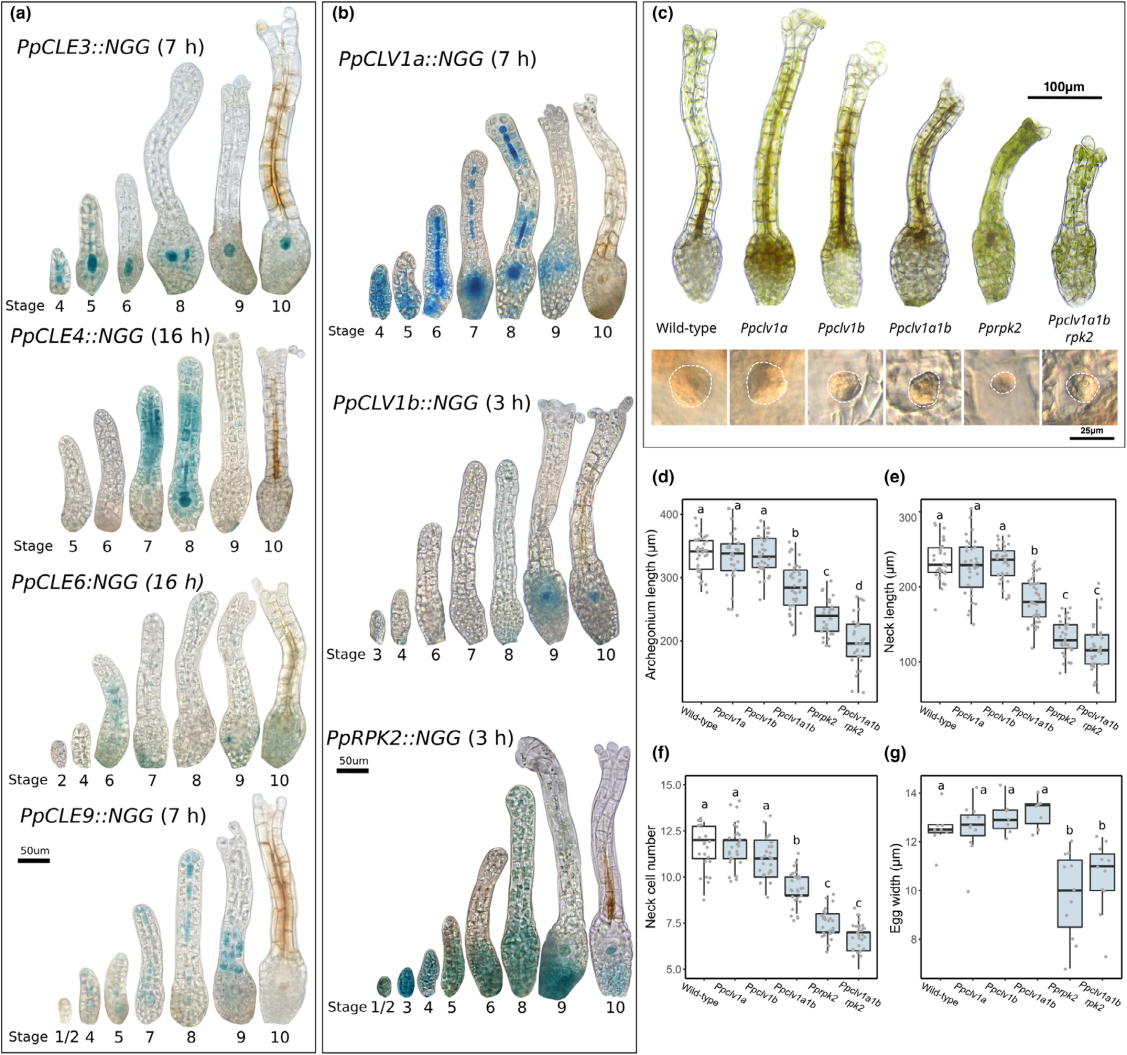
CLAVATA regulates female reproductive development. (a) Light micrographs of Beta‐glucoronidase (GUS)‐stained archegonia from *Physcomitrium patens* at different stages of development showing peptide‐encoding gene promoter activity. *PpCLE3::NGG* activity was detected in inner cells of the neck and the egg cell lineage, *PpCLE4::NGG* activity was detected in the Stages 7 and 8 neck and developing egg, and *PpCLE6::NGG* activity was broad and diffuse. *PpCLE9::NGG* activity marked the tip, inner neck cells and egg cell lineage, and after opening, staining was present at the base of the neck. (b) Light micrographs of archegonia at different stages of development showing receptor‐encoding gene promoter activity. *PpCLV1a::NGG* activity was detected throughout Stage 4 archegonia, at later stages narrowing down to the inner cells of the neck and developing egg. *PpCLV1b::NGG* activity mainly marked egg cells at Stages 9 and 10 of development. *PpRPK2::NGG* activity was present throughout the initiating archegonium (Stages 1–4), later narrowing to the venter (Stages 5 to 10) and developing eggs. Staining times are displayed in brackets. Bar, 50 μm (c) Light micrographs of fully developed archegonia and eggs (white dashed lines) from wild‐type (WT), *Ppclv1a*, *Ppclv1b*, *Ppclv1a1b*, *Pprpk2* and *Ppclv1a1brpk2* mutant plants. Bars: 100 μm (top), 25 μm (bottom). (d) Box plot showing mature archegonium lengths in WT and receptor mutant plants; *n* > 30. (e) Boxplot showing mature archegonial neck lengths in WT and receptor mutant plants; *n* > 30. (f) Boxplot showing the number of cells in the archegonial neck in WT and receptor mutant plants; *n* > 22. (g) Boxplot showing egg width in WT and receptor mutant plants; *n* = 7–11. In all box plots, midlines represent the median value, boxes represent the interquartile range, and whiskers represent 95% confidence intervals. Different letters indicate significant differences between groups identified by ANOVA and Tukey test. *P* < 0.05.

To evaluate the potential contribution of CLAVATA to female reproductive development, we next examined the activity of peptide and receptor‐encoding gene promoters in archegonia at different stages of development (Fig. 5). Archegonia undergo formative cell divisions at Stages 1–5 of development, and cell division and elongation then lengthen the neck at Stages 5–7 (Landberg *et al*., 2013). Cell division ceases at Stage 7, and cells at the apices rupture at Stage 9 to open the hollow neck cavity, which turns brown at Stage 10 (Landberg *et al*., 2013). *PpCLE1::NGG*, *PpCLE2::NGG*, *PpCLE5::NGG*, *PpCLE7::NGG* and *PpCLE8::NGG* activities were undetectable or detected in a minority of samples (Fig. S4). While *PpCLE6::NGG* activity was weak and diffuse through archegonium development, *PpCLE3::NGG*, *PpCLE4::NGG* and *PpCLE9::NGG* activities were strong and consistent in eggs, canal cells or both, with *PpCLE3::NGG* and *PpCLE9::NGG* activities starting at early developmental stages (Fig. 5a). *PpCLE9::NGG* activity was detected in the tip cells of Stages 4 to 7 archegonia, and broad *PpCLE4::NGG* activity in the neck was detected at Stages 7 to 9 of archegonial development (Fig. 5a). Broad *PpCLV1a::NGG* activity was detected up to Stage 4 of archegonium development and later became stronger in the internal egg and canal cell lineage (Fig. 5b). *PpCLV1b::NGG* activity was primarily detected in egg cells at Stages 9 and 10 of development (Fig. 5b). *PpRPK2::NGG* activity was broad up to Stage 4 of archegonium development but was stronger at the base from Stage 5 to Stage 6 and was present in the egg cell at Stages 9 and 10 of development (Fig. 5b). Thus, *PpCLE3::NGG*, *PpCLE4::NGG*, *PpCLE6::NGG, PpCLE9::NGG*, *PpCLV1a::NGG*, *PpCLV1b::NGG* and *PpRPK2::NGG* activities indicated potential roles for CLAVATA peptides and receptors in female reproductive development.

### *PpCLV1a* , *PpCLV1b* and *PpRPK2* regulate female reproductive development

To further investigate such potential roles and validate our expression data, we next analysed receptor mutant phenotypes (Figs 5c–g, S5). While *Ppclv1a* and *Ppclv1b* mutant archegonia resembled those of WT plants, and archegonial initiation and overall structure appeared normal in all mutants, archegonial morphology was perturbed in *Ppclv1a1b*, *Pprpk2* and *Ppclv1a1brpk2* mutants. Mutant archegonia had shorter necks with fewer cells than WT archegonia (Fig. 5c–f), and *Ppclv1a1brpk2* mutants also had reduced overall length (Fig. 5d), showing an additive effect of *Ppclv1a1b* and *Pprpk2* mutations. Mutants also displayed defects in egg development (Figs 5c, S5). Whereas WT, *Ppclv1a*, *Ppclv1b* and *Ppclv1a1b* mutant eggs were similar sizes and spherical, *Pprpk2* and *Ppclv1a1brpk2* mutant eggs were more variable in size but usually smaller than WT eggs (Fig. 5c,g), and sometimes venters contained several small cells with irregular shapes and sizes (Fig. S5). Thus, defects in egg development may underlie *Pprpk2* mutant sterility, but *Ppclv1a1b* mutant sterility (Fig. 3) had no obvious morphological basis in female reproductive development.

### CLE peptides and their receptors are produced in diverse sporophytic tissues

**Fig. 6:**
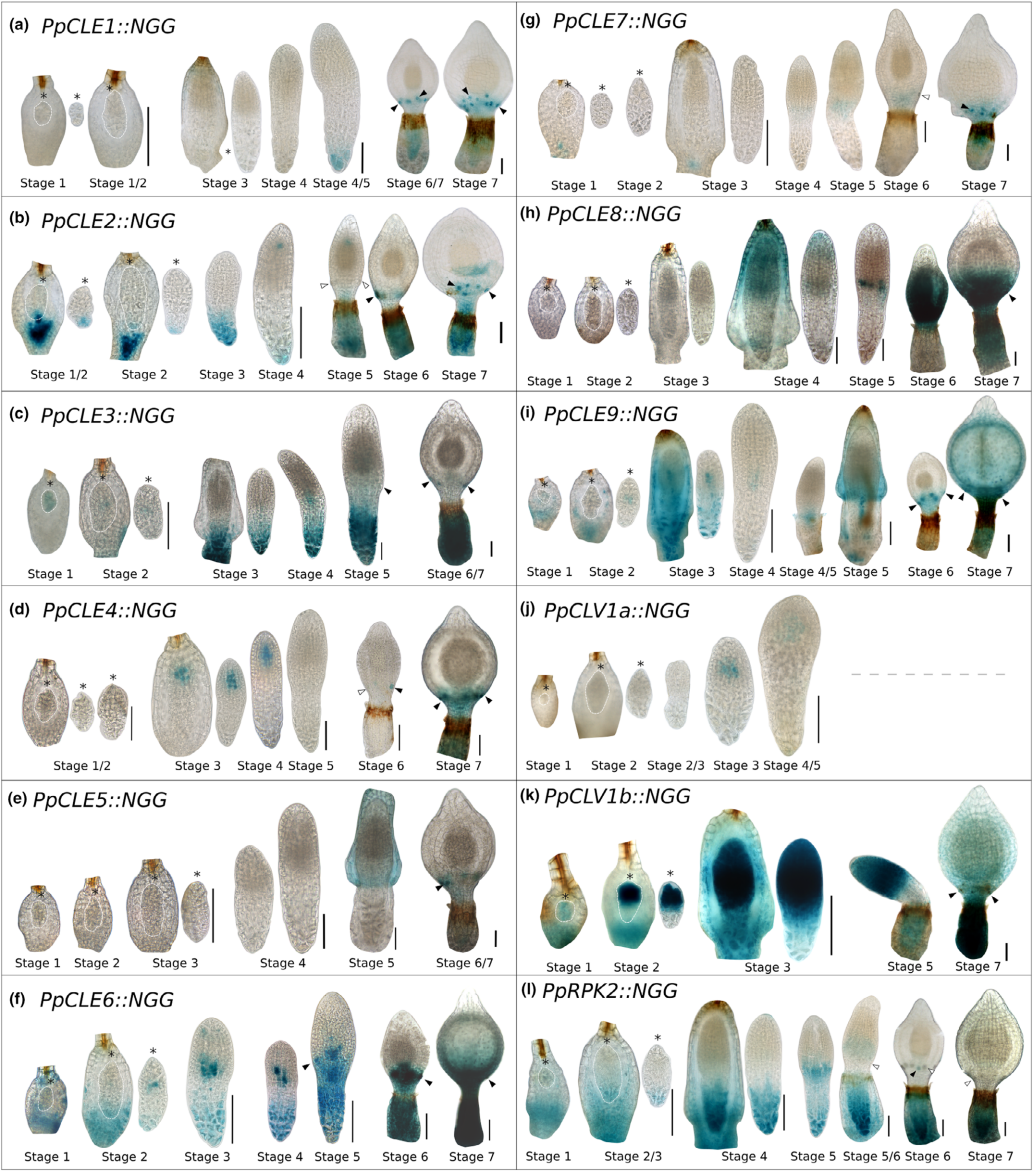
*PpCLE*, *PpCLV1a*, *PpCLV1b* and *PpRPK2* promoters were active in diverse sporophytic tissues in *Physcomitrium patens*. (a) *PpCLE1::NGG* signal was detected at the sporophyte foot and in stomata. (b) *PpCLE2::NGG* signal was detected in maternal tissue, and in the sporophyte foot, stomata and a few apical sporogenic cells. (c) *PpCLE3::NGG* signal was detected in maternal tissue, Stage 1 embryos, and the foot and in stomata of developing sporophytes. (d) *PpCLE4::NGG* signal was detected in sporogenic tissues. (e) *PpCLE5::NGG* activity was detected in stomata. (f) *PpCLE6::NGG* was detected in maternal tissue, the sporophyte foot, stomata and a group of cells in the middle of the sporophyte. (g) *PpCLE7::NGG* signal was detected in maternal tissue, in sporophyte hypophyses and stomata. (h) *PpCLE8::NGG* signal was detected in and around stomata and at the Stage 4 sporophyte tip. (i) *PpCLE9::NGG* signal was detected in maternal tissue, the sporophyte foot, stomata and a file of cells at the centre of the sporophyte. (j) *PpCLV1a::NGG* signal was detected in sporogenic tissue. (k) *PpCLV1b::NGG* activity was present in the apical part of the developing sporophyte. (l) *PpRPK2::NGG* signal was detected in the basal part and a band in the middle of the sporophyte. Staining times were 3 h (*PpCLV1b::NGG*, *PpRPK2::NGG*), 7 h (*PpCLE3::NGG*, *PpCLE9::NGG*, *PpCLV1a::NGG*) or 16 h (*PpCLE1::NGG*, *PpCLE2::NGG*, *PpCLE4::NGG*, *PpCLE5::NGG*, *PpCLE6::NGG* and *PpCLE7::NGG*). Asterisks indicate expected position of apical cell. Arrowheads indicate stomata with (black) or without (white) staining. Bars, 100 μm.

As *Ppclv1a1b*, *Pprpk2* and *Ppclv1a1brpk2* mutants were sterile, it was not possible to identify roles for CLAVATA in sporophyte development by mutant phenotype analysis. However, we were able to identify likely sites of CLAVATA peptide and receptor production using *promoter::NGG* lines (Fig. 6). During sporophyte development, the apical cells first produce lateral merophytes (Stage 1), which then start dividing to make the embryonic axis (Stage 2) (Coudert *et al*., 2019). The apical cell arrests (Stage 3) and an intercalary meristem around the middle of the embryo activates to extend the embryonic axis (Stage 4) (Coudert *et al*., 2019). The apical portion of the sporophyte then swells to form the sporangium (Stage 5), which contains the spore mass and columella and has stomata in a ring around the base (Stage 6; Coudert *et al*., 2019). The capsule becomes spherical at Stage 7 (Coudert *et al*., 2019). At early developmental stages (1–3), *PpCLE3::NGG*, *PpCLE6::NGG* and *PpCLE9::NGG* activities were broad, and *PpCLE2::NGG*, *PpCLE3:NGG*, *PpCLE6:NGG*, *PpCLE7::NGG* and *PpCLE9::NGG* lines showed activity in parental tissue below the sporophyte foot (Fig. 6b,c,f,g,i). *PpCLE7::NGG* and *PpCLE8::NGG* were active in the hypophysis from Stage 4 (Fig. 6g,h), and all nine *PpCLE::NGG* reporters were active in stomata (Fig. 6a–i). *PpCLE1::NGG*, *PpCLE2::NGG*, *PpCLE3::NGG*, *PpCLE6::NGG* and *PpCLE9::NGG* activities were detected in the sporophyte foot (Fig. 6a–c,f,i). Some patterns of promoter activity were gene‐specific; for instance, *PpCLE4::NGG* activity was detected in sporogenic tissue (Fig. 6d), while *PpCLE2::NGG* activity was detected in a few cells at the upper end of the developing spore mass, and *PpCLE6::NGG* activity was detected at the lower end (Fig. 6b,f). *PpCLE3::NGG*, *PpCLE6::NGG, PpCLE7::NGG, PpCLE8::NGG* and *PpCLE9::NGG* activities were detected in groups of cells in the intercalary region, and *PpCLE9::NGG* activity was in apical‐basal cell files in which water‐conducting cells later differentiate (Fig. 6c,f,g–i). In *PpCLV1a::NGG* lines, we were only able to retrieve sporophytes up to Stage 4 of development due to morphological defects likely arising from sustained tissue culture. However, *PpCLV1a::NGG* activity was consistently detected in sporogenic tissues in a similar pattern to *PpCLE4::NGG* activity (Fig. 6j). While *PpCLV1b::NGG* activity was strong in the apical half of the sporophyte (Fig. 6k), *PpRPK2::NGG* activity was strong in the basal sporophyte and later formed a band in the stomatal region (Fig. 6l). Expression was largely nonoverlapping at Stage 2 of sporophyte development, but at Stages 3–5, it appeared juxtaposed or overlapping at the site of the intercalary meristem. Overall, promoter activities suggest the production of multiple CLE peptides in stomata and at the sporophyte foot, and their likely perception by PpCLV1b in the apical and PpRPK2 in the basal sporophyte.

### Different genes are co‐expressed in gametophytes and sporophytes

**Fig. 7:**
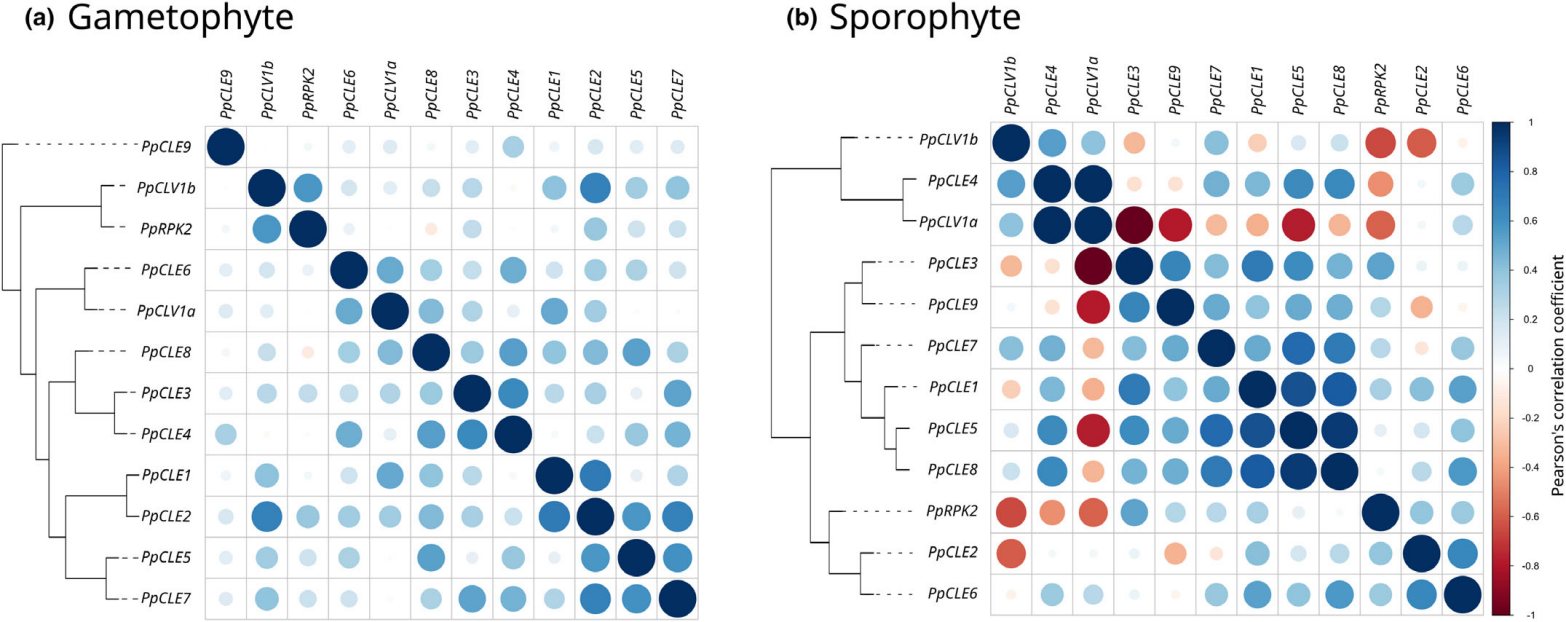
Different *CLAVATA* genes are co‐expressed in gametophytes and sporophytes in *Physcomitrium patens*. Plots represent similarity in gene expression patterns between genes, with 1 : 1 pairwise comparison diagonally crossing the grid at 100% similarity. The colour, intensity and size of each spot represent the similarity in expression patterns between each pair (Pearson's correlation coefficient), with dark blue spots showing the highest degree of similarity and dark red spots the highest dissimilarity. Solid lines in the dendrograms to the left of each diagram depict the degree of similarity between genes based on Pearson's correlation coefficient, with shorter branch distances between genes representing greater similarity. Dashed lines are included to connect branches to gene names. (a) Correlation plot showing that *PpCLE1* and *PpCLE2* have the most similar expression patterns in gametophytes, followed by *PpCLE3* and *PpCLE4*, *PpCLE5* and *PpCLE7* and *PpCLV1b* and *PpRPK2*. (b) Correlation plot showing that *PpCLE4* and *PpCLV1a* have the most similar expression patterns in sporophytes, followed by *PpCLE5*, *PpCLE8* and *PpCLE1*. *PpCLE3* and *PpCLV1a* had the most divergent expression patterns, and *PpCLV1a* and *PpCLV1b* expression patterns were highly divergent from *PpRPK2* expression patterns.

To identify any generalities in our data and further explore potential for co‐expression of peptide and receptor‐encoding gene pairs, we used Pearson's correlation coefficient to analyse the frequencies of gene expression in all tissues examined in this and previous (Nemec‐Venza *et al*., 2022) work (Fig. 7; Table S1). Using data from gametophytes, correlation plots showed that amongst peptide‐encoding genes, *PpCLE1* and *PpCLE2* had the most similar expression patterns, followed by *PpCLE3* and *PpCLE4*, and *PpCLE5* and *PpCLE7* (Fig. 7a). Of the receptor‐encoding genes, *PpCLV1b* and *PpRPK2* had the most similar expression patterns (Fig. 7a). The most obvious potential peptide and receptor pair comprised PpCLE2 and PpCLV1b (Fig. 7a). Analysis of sporophyte expression data identified PpCLE4 and PpCLV1a as a putative peptide and receptor pair, and *PpCLE5*, *PpCLE8* and *PpCLE1* were strongly co‐expressed (Fig. 7b). Of the receptor‐encoding genes, *PpCLV1a* and *PpCLV1b* had the most similar expression patterns (Fig. 7b). Many genes had highly divergent expression patterns, and this divergence was not detected in gametophyte data. For instance, *PpCLV1a* expression patterns were strongly divergent from *PpCLE1*, *PpCLE3*, *PpCLE5*, *PpCLE7*, *PpCLE8* and *PpCLE9* expression patterns, and the *PpCLV1b* expression pattern was divergent from *PpCLE1*, *PpCLE2*, *PpCLE3* and *PpRPK2* expression patterns (Fig. 7b). This analysis showed no strict coupling between peptide and receptor‐encoding gene expression patterns and that different genes are co‐expressed in gametophytes and sporophytes.

## Discussion

### *PpCLEs* likely have divergent functions in moss development

**Fig. 8:**
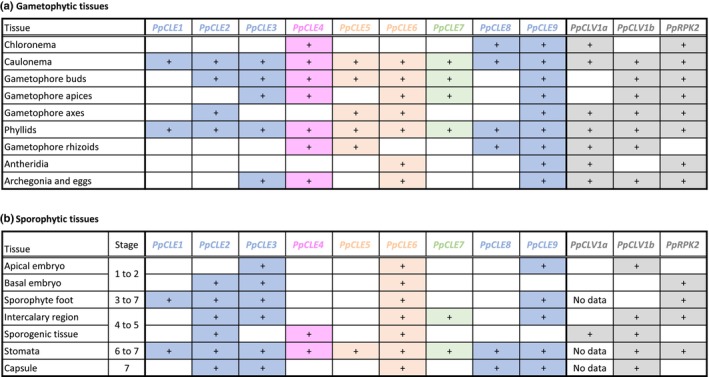
Summary of promoter activities recorded in this study and previous work in *Physcomitrium patens* (Nemec‐Venza *et al*., 2022). (a) In gametophytes, all promoters were found to be active in caulonema and phyllids, whereas other tissues expressed a subset of *PpCLE*s. While *PpCLE9* was expressed in all tissue classes, other promoter activities were detected in a narrower range of tissues. (b) In sporophytes, all promoters were found to be active in stomata, whereas other tissues expressed a subset of *PpCLE*s or receptor‐encoding genes. While *PpCLE6* was expressed in all tissue classes, the promoter activities of other CLAVATA pathway genes were more restricted. Promoter activity was scored as present (+) in which it was detected in 50% or more of samples evaluated in a given tissue class; observed frequencies from specific tissue classes are recorded in Supporting Information Table S1. No data were recorded in some *PpCLV1a::NGG* tissues due to developmental defects in these lines. *PpCLE*s encoding the same presumptive peptide are shaded using the same colour.

CLAVATA pathway genes diversified independently from low ancestral copy numbers in mosses and flowering plants (Whitewoods *et al*., 2018). While 32 CLEs and their receptors function in diverse tissues and organs during Arabidopsis development (Cock & McCormick, 2001; Jun *et al*., 2010; Narasimhan & Simon, 2022) and roles for CLAVATA in moss protonema and gametophore development were previously identified (Whitewoods *et al*., 2018, 2020; Cammarata *et al*., 2022; Nemec‐Venza *et al*., 2022), specific sites of moss CLAVATA activity were previously unknown. Here, we used a comprehensive analysis of *PpCLE*, *PpCLV1a, PpCLV1b* and *PpRPK2* promoter activities throughout *P. patens* development to explore such specificity and guide future work. No *PpCLE* was uniquely expressed in any single tissue type, but patterns of promoter activity were restricted to individual cell types within tissues; for instance, *PpCLE3* promoter activity marked gametophore apical cells and egg cells, *PpCLE5* activity marked rhizoid initiating bud cells, and *PpCLE9* activity marked phyllid midribs. No *PpCLE*s or receptor‐encoding genes showed identical expression patterns, and subsets of *PpCLE*s and receptor promoters were active in each cell or tissue type, with no tissues showing no promoter activity (Fig. 8; Table S1). *PpCLE1‐3*, *PpCLE8* and *PpCLE9* are predicted to encode the same peptide (RMVPTGPNPLHN coloured blue in Fig. 8), and promoter activity indicated that the peptide is likely produced in all tissues. Similarly, *PpCLE5* and *PpCLE6* encode a single peptide (RLVPTGPNPLHN coloured orange in Fig. 8) that is likely produced in all tissues. By contrast, *PpCLE4* and *PpCLE7* encode unique presumptive peptides (respectively RMVPSGPNPLHN and RVVPTGPNPLHN, coloured pink or green in Fig. 8), and their promoters were active in fewer gametophytic (*PpCLE7*) and sporophytic tissues (*PpCLE4 and PpCLE7*) These genes are therefore likely to have more restricted functions (Fig. 8; Table S1). Overall, *PpCLE* genes encoding the same peptide did not have similar promoter activities (Figs 7, 8), and our data therefore indicate that *PpCLE* regulatory regions diversified independently from PpCLE peptide sequences, and in mosses and flowering plants. They are consistent with the notion from other species that promoter evolution generates new CLE signalling sites and might drive divergence of gene function (Hobe *et al*., 2003; Furumizu & Aalen, 2023).

### Putative mechanisms for signalling specificity

Coupled with overlapping receptor expression patterns and the potential for peptides to move, the production of some CLE peptides in many cell and tissue types in *P. patens* (Fig. 8) raises questions about how signalling specificity could arise. In Arabidopsis, such specificity arises from spatially restricted expression of *CLE*s (Jun *et al*., 2010), cell type‐specific receptor combinations (Qian *et al*., 2018; Crook *et al*., 2020; Zhang *et al*., 2024), concentration‐dependent responses to peptides (Pallakies & Simon, 2014) and differential affinities between peptides and receptors (Li *et al*., 2017). In many *P. patens* tissues, *PpCLV1a*, *PpCLV1b* and *PpRPK2* promoter activities were broader than *PpCLE* promoter activities, suggesting that spatially restricted *PpCLE* expression could provide signalling specificity. For instance, *PpCLV1b* and *PpRPK2* promoters were active in overlapping domains in gametophore bud apices, while the *PpCLE3* promoter was active in apical cells (Fig. 1). In antheridia, *PpRPK2* and *PpCLV1a* promoters were broadly active, and *PpCLE6* and *PpCLE9* promoter activities, respectively, co‐localized in the stalk or the antheridial apical cell, implicating *PpCLE6* and *PpCLE9* in antheridium length regulation (Fig. 4). At different stages of archegonium development, *PpCLV1a* and *PpRPK2* promoter activities overlapped with *PpCLE4* promoter activity in the archegonial neck, suggesting that *PpCLE4* may act in neck elongation. There was strong *PpCLE3* promoter activity in egg cells, implicating *PpCLE3* as a partner in generating the observed sterility defects of receptor mutants (Figs 3, 5). Overlapping promoter activities of *PpCLV1b* and *PpRPK2* in gametophore apices and axes (Fig. 2), but exclusive activities in developing sporophytes (Fig. 6), demonstrate that cells can express different receptor combinations, and receptor mutant phenotypes in protonemata (Nemec‐Venza *et al*., 2022), gametophores (Whitewoods *et al*., 2018, 2020; Cammarata *et al*., 2022), phyllids (Fig. S3) and antheridia (Fig. 4) demonstrate that receptors can have specialized functions. Peptide concentration‐dependent responses have been observed in phyllids in which exogenous CLV3 peptide treatment suppresses cell proliferation (Whitewoods *et al*., 2018) and suppresses vein development in *Ppcr4* receptor mutants (Shumbusho *et al*., 2024), but endogenous peptides promote cell proliferation (Fig. S3; Shumbusho *et al*., 2024). Moss peptide binding affinities have not yet been assayed, but this is a further potential mechanism for specificity. Thus, many aspects of CLAVATA function bring potential for signalling specificity and moss cell type diversification during evolution (Kofuji & Hasebe, 2014).

### Sites of receptor expression overlap with auxin sensing minima in multiple developmental contexts

A notable feature of the data we collected was that many sites of *CLAVATA* gene expression overlapped with previously identified sites of hormone signalling. For instance, *PpRPK2* acts in parallel with cytokinin to regulate gametophore bud development, and independently from *PpCLV1a/PpCLV1b* (Cammarata *et al*., 2022) and *PpRPK2* and *PpCLV1a/PpCLV1b* both regulate auxin synthesis and auxin transporter (*PpPIN A‐C*) expression to determine plant spread in protonemata (Nemec‐Venza *et al*., 2022). The data presented here identify caulonemal apical cells, gametophore apical cells, the base of developing leaves, rhizoid tips, and pre‐egg and egg cells as regions of high *PpRPK2* and *PpCLV1a/PpCLV1b* promoter activity, coinciding with previously identified sites of minimal auxin sensing (Thelander *et al*., 2019; Landberg *et al*., 2021). Moreover, *PpPIN* expression domains are similar to *CLAVATA* expression domains in leaves and reproductive tissues (Landberg *et al*., 2013; Viaene *et al*., 2014), and *Pppin* mutants have fertility defects (Bennett *et al*., 2014; Lüth *et al*., 2023), a phenotype shared by CLAVATA receptor mutants (Figs 3, 4, 5). Auxin synthesis is required for normal egg development, and biosynthetic *Ppshi*‐*2* mutant eggs resemble *Pprpk2* mutant eggs (Landberg *et al*., 2013). These data suggest that CLAVATA receptor signalling could modulate auxin homeostasis in multiple developmental contexts in *P. patens*, and divergent receptor mutant phenotypes show that *PpRPK2* and *PpCLV1a/PpCLV1b* act independently in reproductive development as well as vegetative development (Cammarata *et al*., 2022). Identifying upstream hormonal and environmental regulators of receptor signalling and downstream transcription factors that modulate hormone homeostasis remains a key challenge for future work.

### It is not clear whether CLAVATA ancestrally promoted or suppressed meristematic proliferation

Recent papers have led to a paradigm that the ancestral function of CLAVATA signalling in land plants is in the promotion of meristematic stem cell proliferation (Hirakawa, 2022; Furumizu & Shinohara, 2024; Renninger *et al*., 2024). This paradigm is based on results from Arabidopsis sporophyte (Fletcher *et al*., 1999) and fern (*C. richardii*) (Renninger *et al*., 2024) and liverwort (*M. polymorpha*) (Hirakawa *et al*., 2020) gametophyte meristems, which are nonhomologous structures (Fouracre & Harrison, 2022). Moreover, previous work in *P. patens* has shown that CLAVATA suppresses proliferation in moss gametophore meristems (Whitewoods *et al*., 2018, 2020; Cammarata *et al*., 2022). CLAVATA can repress or promote cell proliferation depending on the developmental context in Arabidopsis (Crook *et al*., 2020; Zhang *et al*., 2024) and moss (Fig. S3; Whitewoods *et al*., 2018). Taken together, these data support the inference that there is an ancestral role for CLAVATA in meristematic proliferation in land plants but do not allow inference of the direction of regulation. Mosses and liverworts are sister lineages in the Setaphyte clade (Puttick *et al*., 2018; Harris *et al*., 2020), and hence, it is not clear whether CLAVATA promoted or suppressed meristematic cell proliferation in the ancestral Setaphyte. Moreover, functions in hornwort and lycophyte gametophytes are unknown, and these taxon sampling gaps make it impossible to infer the ancestral direction of meristematic CLAVATA function in land plant gametophytes.

Thus far, sporophyte meristem functions have only been directly tested in flowering plants, and there are many unknowns in relation to sporophyte meristem evolution (Fouracre & Harrison, 2022). While the central stem cell and proliferative meristem zone configuration is conserved to the level of vascular plants, bryophyte sporophytes have proliferative zones in an intercalary region of the axis (Fouracre & Harrison, 2022). *KNOX* genes are conserved regulators of meristem proliferation in vascular plants (Harrison *et al*., 2005; Jasinski *et al*., 2005; Yanai *et al*., 2005; Ambrose & Vasco, 2016), and intercalary proliferation is also controlled by *KNOX* gene function in *P. patens* and *M. polymorpha* (Sakakibara *et al*., 2008; Singer & Ashton, 2008; Coudert *et al*., 2019; Dierschke *et al*., 2021), indicating likely homology between rapidly proliferative meristem zones (Fouracre & Harrison, 2022). However, amongst bryophytes, only mosses have stem cells in sporophyte apices (Fouracre & Harrison, 2022). The data we present here provide the first comparison of CLAVATA activities between angiosperm and bryophyte sporophyte meristems. Given that we detected no apical cell‐specific *PpCLE* or receptor‐like promoter activity, they suggest that CLAVATA is unlikely to regulate moss sporophyte stem cell function. However, *PpCLV1b* and *PpRPK2* promoter activities overlap, and multiple CLEs are produced in the intercalary meristem, suggesting that CLAVATA may regulate meristematic cell proliferation. Thus, our data support the hypothesis that vascular plant meristems and bryophyte intercalary meristems may be homologous and identify potential conservation in meristematic gene regulatory networks.

## Competing interests

None declared.

## Author contributions

All authors contributed to the design of the study. ZN‐V and GRLG undertook the experimental work and data analyses with supervision from CJH. All authors contributed to manuscript preparation, editing and revision.

## Disclaimer

The New Phytologist Foundation remains neutral with regard to jurisdictional claims in maps and in any institutional affiliations.

## Supporting information


**Fig. S1** *CLAVATA* promoter activities not included in Fig. 1.
**Fig. S2** Validation of GUS results with GFP fluorescence.
**Fig. S3** Characterization of phyllid phenotypes in *Ppclv1a, Ppclv1b, Ppclv1a1b* and *Pprpk2* mutants.
**Fig. S4** GUS‐stained gametangia from lines showing low or no expression.
**Fig. S5** Egg cells images from fresh tissue.
**Table S1** CLAVATA promoter activities recorded in this study and previous work.
Please note: Wiley is not responsible for the content or functionality of any Supporting Information supplied by the authors. Any queries (other than missing material) should be directed to the *New Phytologist* Central Office.

## Data Availability

The data that supports the findings of this study are available in the main text and Supporting Information in Figs S1–S5 and Table S1 of this article.
