# Supplementary material for: Diversification of CLE expression patterns and nonmeristematic roles for CLAVATA receptor‐like kinases in a moss
Source: New Phytol. 2025 May 6;247(1):325–40. doi: 10.1111/nph.70170 (PMC12138186; doi:10.1111/nph.70170)
Supplement: Supplementary file 1 — Fig. S1 CLAVATA promoter activities not included in Fig. 1. Fig. S2 Validation of GUS results with GFP fluorescence. Fig. S3 Characterization of phyllid phenotypes in Ppclv1a, Ppclv1b, Ppclv1a1b and Pprpk2 mutants. Fig. S4 GUS‐stained gametangia from lines showing low or no expression. Fig. S5 Egg cells images from fresh tissue. Table S1 CLAVATA promoter activities recorded in this study and previous work. Please note: Wiley is not responsible for the content or functionality of any Supporting Information supplied by the authors. Any queries (other than missing material) should be directed to the New Phytologist Central Office. [file NPH-247-325-s001.pdf]

New Phytologist Supporting Information

Article title: Diversification of CLE expression patterns and non-meristematic roles for CLAVATA receptor-like kinases in a moss.

Authors: Zoe Nemec-Venza, George R. L. Greiff and C. Jill Harrison

Article acceptance date: 3 April 2025

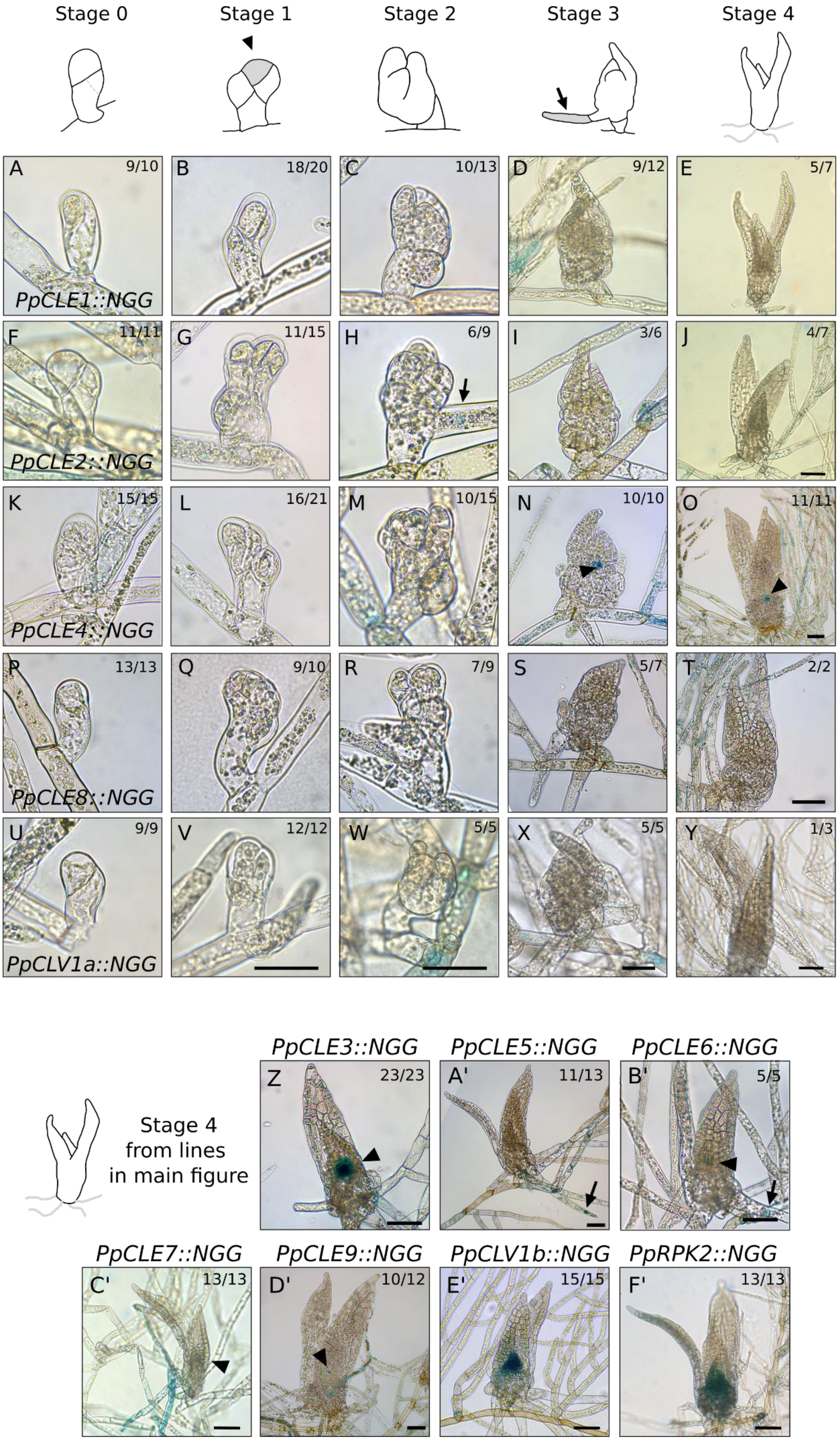

Stage 0
Stage 1
Stage 2
Stage 3
Stage 4
A 9/10
PpCLE1::NGG
B 18/20
C 10/13
D 9/12
E 5/7
F 11/11
PpCLE2::NGG
G 11/15
H 6/9
I 3/6
J 4/7
K 15/15
PpCLE4::NGG
L 16/21
M 10/15
N 10/10
O 11/11
P 13/13
PpCLE8::NGG
Q 9/10
R 7/9
S 5/7
T 2/2
U 9/9
PpCLV1a::NGG
V 12/12
W 5/5
X 5/5
Y 1/3
Stage 4 from lines in main figure
PpCLE3::NGG
Z 23/23
PpCLE5::NGG
A' 11/13
PpCLE6::NGG
B' 5/5
PpCLE7::NGG
C' 13/13
PpCLE9::NGG
D' 10/12
PpCLV1b::NGG
E' 15/15
PpRPK2::NGG
F' 13/13

Supplementary Figure 1: *CLAVATA* promoter activities not included in Figure 1. (A-E) The *PpCLE1* promoter was usually inactive at **(A)** Stage 0, **(B)** Stage 1, **(C)** Stage 2, **(D)** Stage 3 and **(E)** Stage 4 of gametophore development. **(F-J)** *PpCLE2::NGG* was usually inactive at **(F)** Stage 0, **(G)** Stage 1, **(H)** Stage 2, **(I)** Stage 3 and **(J)** Stage 4 of gametophore development. *PpCLE2::NGG* activity was evident in rhizoids from Stage 2. **(K-O)** *PpCLE4::NGG* was usually inactive at **(K)** Stage 0, **(L)** Stage 1, **(M)** Stage 2, **(N)** Stage 3 and **(O)** Stage 4 of gametophore development. **(P-T)** *PpCLE8::NGG* was inactive at **(P)** Stage 0, **(Q)** Stage 1, **(R)** Stage 2, **(S)** Stage 3 and **(T)** Stage 4 of gametophore development. **(U-Y)** *PpCLV1a::NGG* was inactive at **(U)** Stage 0, **(V)** Stage 1, **(W)** Stage 2, **(X)** Stage 3 and **(Y)** Stage 4 of gametophore development. **(Z-F')** Stage 4 buds from **(Z)** *PpCLE3::NGG,* **(A')** *PpCLE5::NGG,* **(B')** *PpCLE6::NGG,* **(C')** *PpCLE7::NGG,* **(D')** *PpCLE9::NGG,* **(E')** *PpCLV1b::NGG* and **(F')** *RPK2::NGG* lines. Arrowheads indicate apical signal, arrows indicate rhizoid signal. Numbers indicate the proportion of buds with a similar expression pattern. Scale bars at Stages 0-2 = 50 µm, Scale bars at Stages 3-4 = 100 µm.

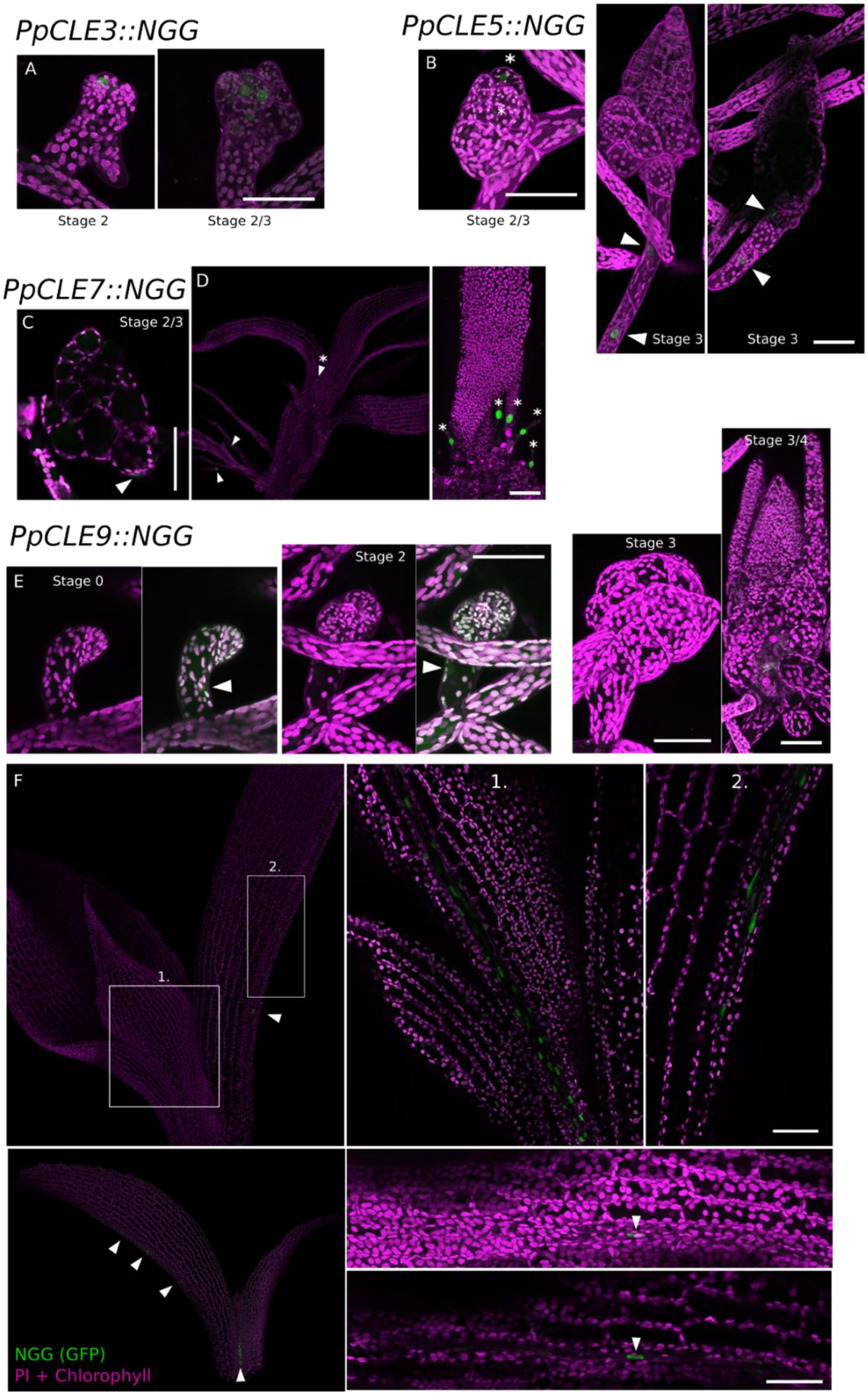

PpCLE3::NGG
A
Stage 2
Stage 2/3
PpCLE5::NGG
B
Stage 2/3
Stage 3
Stage 3
PpCLE7::NGG
C
Stage 2/3
D
PpCLE9::NGG
E
Stage 0
Stage 2
Stage 3
Stage 3/4
F
1.
2.
NGG (GFP)
PI + Chlorophyll

Supplementary Figure 2: Validation of GUS results with GFP fluorescence. **(A)** Buds from *PpCLE3::NGG* lines showing fluorescence in the apical cell and recent daughters. **(B)** Buds from *PpCLE5::NGG* lines showing signal in an apical hair and rhizoids. **(C)** Buds and **(D)** gametophores from *PpCLE7::NGG* lines showing signal in apical hairs and rhizoids. **(E)** Buds from *PpCLE9::NGG* lines showing faint signal at Stages 0 and 2 of development. The brightness of the GFP channel was increased in the second image of Stage 0 and Stage 2 buds. **(F)** Gametophores apices from *PpCLE9::NGG* lines showing signal in phyllid midribs. Arrowheads indicate nuclear GFP signal. Asterisks indicate hairs. Scale bar = 50 µm.

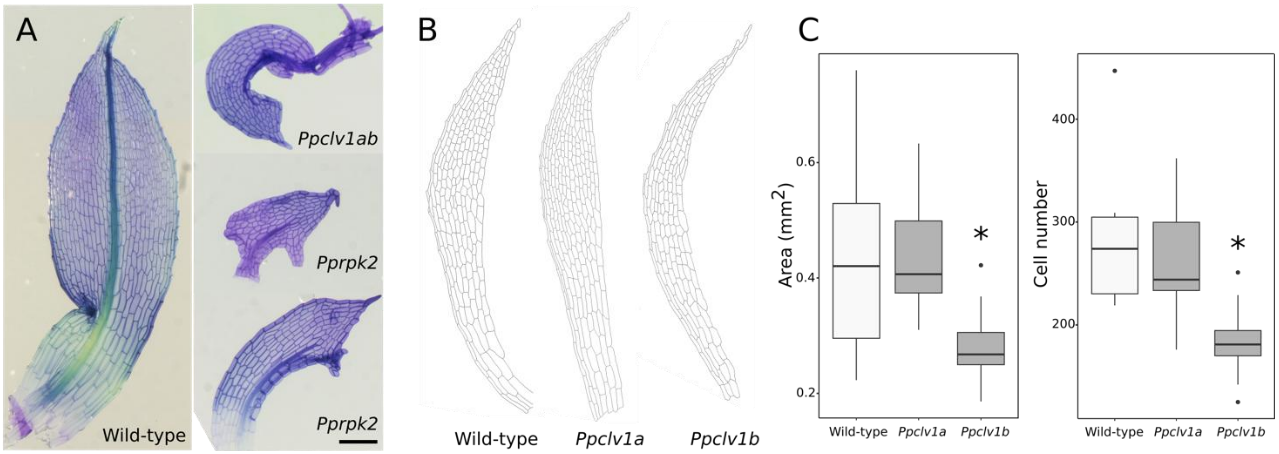

Supplementary Figure 3: Characterization of phyllid phenotypes in *Ppclv1a, Ppclv1b, Ppclv1a1b* and *Pprpk2* mutants. A) Light micrographs of toluidine blue stained phyllids (wild-type phyllid 9 and mutant phyllid 3 from leaf series), showing severe mutant phenotypes excluding *Ppclv1a1b* and *Pprpk2* mutants from quantitative analysis of leaf cell proliferation. Scale bar = 200 µm. **B)** Segmented cell outlines used to quantify cell numbers in half-phyllids from wild-type, *Ppclv1a* and *Ppclv1b* mutant plants (not to scale). **C)** Graphs showing areas and cell numbers of half phyllids from the ninth position in wild-type, *Ppclv1a* and *Ppclv1b* mutant leaf series (n = 10). *Ppclv1b* phyllids were smaller and had fewer cells than wild-type phyllids. * = significantly different from wild-type, p value < 0.05.

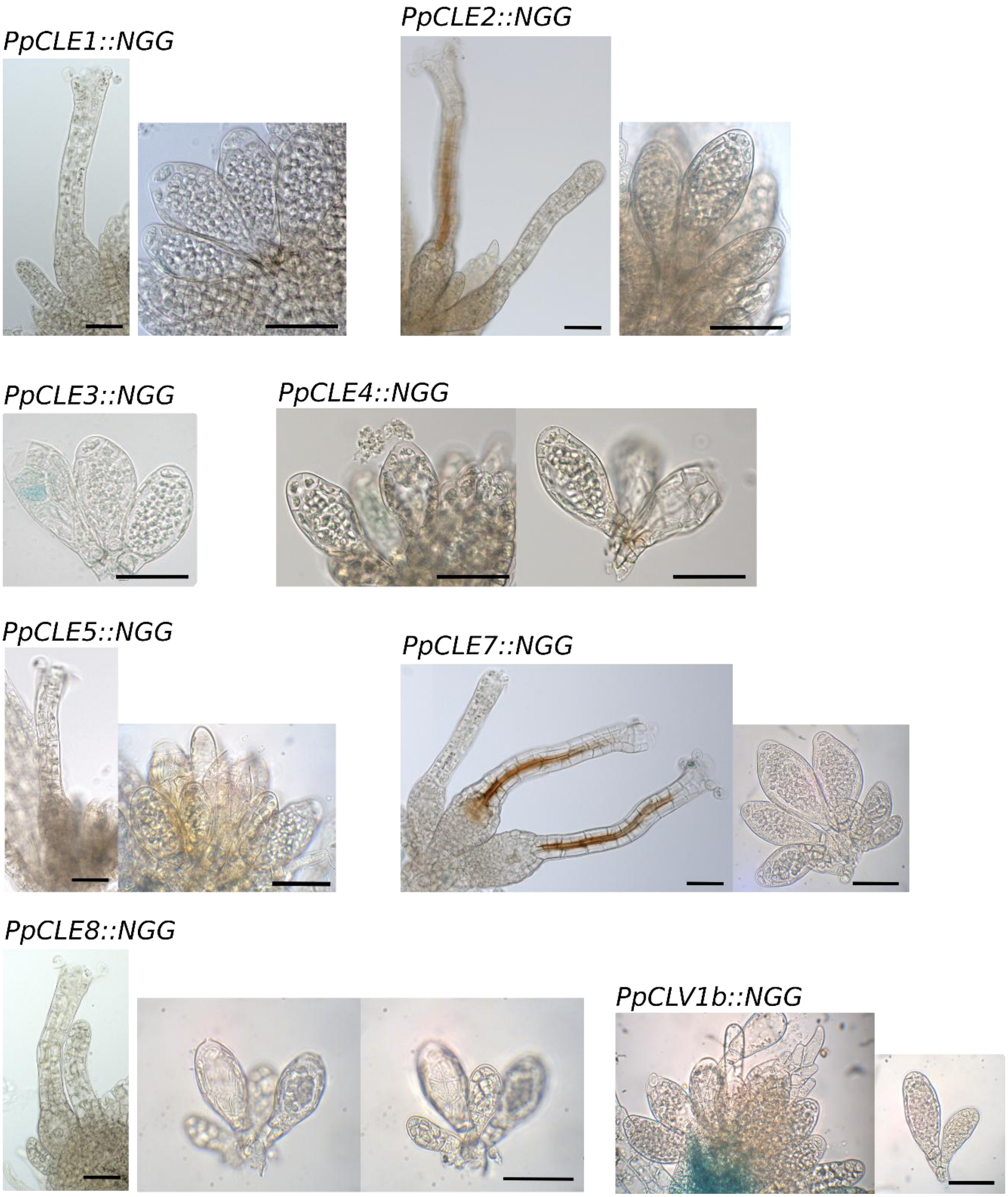

Supplementary Figure 4: **GUS-stained gametangia from lines showing low or no expression.** Less than 50% of antheridia in *PpCLE1::NGG*, *PpCLE2::NGG*, *PpCLE3::NGG*, *PpCLE4::NGG*, *PpCLE5::NGG*, *PpCLE7::NGG*, *PpCLE8::NGG* and *PpCLV1b::NGG* samples showed expression. Less than 50% of archegonia from *PpCLE1::NGG*, *PpCLE2::NGG*, *PpCLE5::NGG*, *PpCLE7::NGG* and *PpCLE8::NGG* lines showed signal. Scale bar = 50 µm

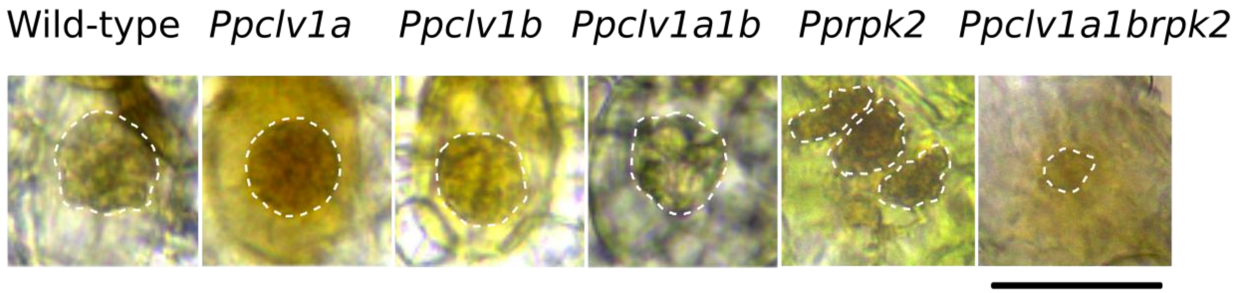

Supplementary Figure 5: Egg cells images from fresh tissue showing the *Pprpk2* multiple egg phenotype. Dashed lines highlight the outline of egg cells. Scale bar = 25 µm.

Tissue class for Table 1	Classes scored for similarity plot	*PpCLE1*	*PpCLE2*	*PpCLE3*	*PpCLE4*	*PpCLE5*	*PpCLE6*	*PpCLE7*	*PpCLE8*	*PpCLE9*	*PpCLV1a*	*PpCLV1b*	*PpRPK2*
**Chloronema**	Germinating spore	0.00	0.03	0.14	0.00	0.02	0.09	0.03	0.10	0.09	**0.58**	0.03	0.03
	Primary chloronema	0.30	0.00	0.13	0.27	0.10	0.21	0.08	**0.50**	**0.63**	**1.00**	0.11	0.19
	Chloronema branches	0.13	0.27	0.33	**0.50**	0.46	0.31	0.46	0.00	**0.70**	0.11	0.00	**0.79**
**Caulonema**	Primary caulonema	**0.53**	**0.69**	**0.80**	**0.83**	**0.87**	**0.86**	**1.00**	**0.63**	**0.88**	**1.00**	**1.00**	**0.97**
	Caulonema tips	0.09	**0.76**	0.46	**0.52**	**0.94**	**0.51**	**0.91**	0.19	**0.91**	0.12	**0.63**	**1.00**
**Gametophore buds**	Cell under the bud (Stage 0-1)	0	0.21	0.08	0.27	**0.58**	**0.8**	**0.68**	0.09	0.32	0.09	**0.57**	**0.73**
	Stage 0	0.10	0.00	0.06	0.00	0.10	0.00	0.20	0.00	**0.67**	0.00	**0.63**	**0.92**
	Stage 1 apical	0.13	0.00	**0.59**	0.24	0.06	0.00	0.21	0.10	0.31	0.00	0.43	**0.74**
	Stage 1 basal	0.00	0.27	0.00	0.05	0.25	0.00	0.21	0.00	0.13	0.00	**0.70**	**0.63**
	Stage 2-4 apex	0.00	0.00	**1.00**	**0.89**	0.00	0.35	**0.88**	0.32	**0.53**	0.00	0.00	0.00
	Stage 2-4 broader apex	0.25	0.09	0.00	0.00	0.13	0.00	0.00	0.00	0.00	0.00	**0.93**	**0.86**
	Stage 2-4 rhizoid	0.44	**0.59**	0.07	0.28	**0.87**	0.38	**0.83**	0.21	0.47	0.00	0.29	**0.71**
**Gametophore apex**	Gametophore apex	0.00	0.44	**1.00**	**1.00**	0.37	**1.00**	**0.90**	0.05	**1.00**	0.38	**0.85**	**1.00**
**Gametophore axis**	Gametophore axis	0.04	**0.59**	0.06	0.03	**0.50**	**1.00**	0.24	0.27	**0.93**	**1.00**	**1.00**	**0.85**
**Phyllids**	Phyllid lamina	**1.00**	**1.00**	**1.00**	0.00	0.00	0.00	**1.00**	0.00	**0.50**	**1.00**	**1.00**	**1.00**
	Phyllid base	**1.00**	**1.00**	**1.00**	**1.00**	**0.50**	**1.00**	**0.50**	**1.00**	**0.50**	**1.00**	**1.00**	**1.00**
	Phyllid midrib	**1.00**	**0.50**	0.00	0.00	0.00	**1.00**	0.00	0.00	**1.00**	**1.00**	**0.50**	0.20
**Gametophore rhizoids**	Gametophore rhizoids	0.11	0.44	0.32	**0.56**	**1.00**	0.41	0.38	**0.64**	**0.52**	**0.52**	**0.52**	0.41
**Antheridia**	Antheridia (Stage 1-5 apex)	0.00	0.00	0.00	0.19	0.00	0.00	0.00	0.03	**1.00**	**0.53**	0.13	**0.98**
	Antheridia (Stage 1-5 base)	0.00	0.00	0.42	0.19	0.00	**1.00**	0.00	0.03	0.00	**0.97**	0.13	**0.98**
	Antheridia (Stage 6-8 apex)	0.24	0.02	0.44	0.41	0.29	0.00	0.20	0.30	**1.00**	0.40	0.04	0.30
	Antheridia (Stage 6-8 base)	0.24	0.02	0.44	0.41	0.29	**1.00**	0.20	0.30	0.00	**0.90**	0.04	0.30
**Archegonia and eggs**	Egg and canal cells (Stage 4-8)	0.00	0.00	**0.96**	**0.77**	0.14	**0.64**	0.00	0.10	**0.95**	**1.00**	0.07	**0.91**
	Eggs at (Stage 9-10)	0.00	0.00	**0.75**	0.10	0.08	0.20	0.00	0.00	**0.62**	0.00	**0.90**	**1.00**
	Archegonium tip	0.00	0.00	0.00	0.00	0.00	0.00	0.00	0.00	**1.00**	0.25	0.00	**0.50**
	Archegonium neck (Stage 7-9)	0.00	0.00	0.00	**0.77**	0.00	**0.71**	0.00	0.00	**1.00**	0.00	0.00	0.30
	Archegonium venter	0.00	0.15	0.00	0.10	0.00	0.30	0.00	0.00	0.10	0.00	0.20	**0.97**
**Sporophyte development**	Maternal tissue	0.46	**1.00**	**0.63**	0.00	0.27	**1.00**	0.14	0.40	**1.00**	0.00	0.00	**1.00**
**Apical embryo**	Apical embryo (Stage 1-2)	0.00	0.00	**0.67**	0.00	0.09	**0.50**	0.00	0.00	**1.00**	0.00	**1.00**	0.00
**Basal embryo**	Basal embryo (Stage 1-2)	0.18	**1.00**	**0.67**	0.00	0.18	**0.67**	0.00	0.00	0.33	0.00	0.00	**0.67**
**Sporophyte foot**	Sporophyte foot (Stage 3-7)	**0.60**	**0.96**	**0.95**	0.00	0.21	**1.00**	0.00	0.14	**0.52**	**No data**	0.00	**1.00**
**Intercalary region**	Intercalary region (Stage 4-5)	0.00	**0.50**	**0.63**	0.00	0.17	**0.93**	**0.67**	0.13	**0.77**	0.00	**1.00**	**1.00**
**Sporogenic tissue**	Sporogenic tissue (Stage 3-5)	0.00	**0.67**	0.00	**0.57**	0.00	**0.90**	0.00	0.00	0.00	**1.00**	**1.00**	0.00
**Stomata**	Stomata (Stage 6-7)	**1.00**	**0.71**	**1.00**	**0.80**	**1.00**	**1.00**	**0.92**	**1.00**	**1.00**	**No data**	**1.00**	**0.50**
**Capsule**	Capsule (Stage 7)	0.25	**1.00**	**0.50**	0.30	0.31	**1.00**	0.08	**0.50**	**0.50**	**No data**	**1.00**	0.00

Supplementary Table 1: CLAVATA promoter activities recorded in this study and previous work in *P. patens* (Nemec-Venza *et al.*, 2022)**.** The frequency at which signal was detected in specified cells and tissue types is shown. Where the frequency was 50% or more, promoter activity was scored as present for Table 1 shown in the main text, and bold lines delimit tissue classes used in the summary table. No data were recorded in some *PpCLV1a::NGG* tissues due to developmental defects in these lines. *PpCLE*s encoding the same presumptive peptide are shaded using the same colour.

References from Supporting Information:

Nemec-Venza Z, Madden C, Stewart A, Liu W, Novák O, Pencik A, Cuming AC, Kamisugi Y, Harrison CJ. 2022. CLAVATA modulates auxin homeostasis and transport to regulate stem cell identity and plant shape in a moss. *New Phytologist* **234**: 149-163.
